# Changing and stable chromatin accessibility supports transcriptional overhaul during neural stem cell activation and is altered with age

**DOI:** 10.1111/acel.13499

**Published:** 2021-10-23

**Authors:** Sun Y. Maybury‐Lewis, Abigail K. Brown, Mitchell Yeary, Anna Sloutskin, Shleshma Dhakal, Tamar Juven‐Gershon, Ashley E. Webb

**Affiliations:** ^1^ Department of Molecular Biology, Cell Biology, and Biochemistry Brown University Providence Rhode Island USA; ^2^ The Mina and Everard Goodman Faculty of Life Sciences Bar‐Ilan University Ramat Gan Israel; ^3^ Carney Institute for Brain Science Brown University Providence Rhode Island USA; ^4^ Center on the Biology of Aging Brown University Providence Rhode Island USA

**Keywords:** aging, chromatin accessibility, neural stem cells, stem cell activation

## Abstract

Neural stem cells (NSCs) in the adult and aged brain are largely quiescent, and require transcriptional reprogramming to re‐enter the cell cycle. However, the mechanisms underlying these changes and how they are altered with age remain undefined. Here, we identify the chromatin accessibility differences between primary neural stem/progenitor cells in quiescent and activated states. These distinct cellular states exhibit shared and unique chromatin profiles, both associated with gene regulation. Accessible chromatin states specific to activation or quiescence are active enhancers bound by key pro‐neurogenic and quiescence factors. In contrast, shared sites are enriched for core promoter elements associated with translation and metabolism. Unexpectedly, through integrated analysis, we find that many sites that become accessible during NSC activation are linked to gene repression and associated with pro‐quiescence factors, revealing a novel mechanism that may preserve quiescence re‐entry. Furthermore, we report that in aged NSCs, chromatin regions associated with metabolic and transcriptional functions bound by key pro‐quiescence transcription factors lose accessibility, suggesting a novel mechanism of age‐associated NSC dysfunction. Together, our findings reveal how accessible chromatin states regulate the transcriptional switch between NSC quiescence and activation, and how this switch is affected with age.

## Graphical abstract

Quiescent neural stem cells in the adult mammalian brain overhaul their transcriptome as they activate for proliferation and neurogenesis. We report that distinct modes of chromatin dynamics at promoters and enhancers support stem cell activation, and that key metabolic genes become less accessible with age.

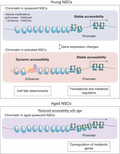

## 1 INTRODUCTION

Neural stem cells (NSCs) are the source of new neurons, astrocytes, and oligodendrocytes in the adult mammalian brain. Studies in rodents show that adult‐born neurons contribute to learning and memory, sensory functions, and mood regulation (Bond et al., 2015). NSCs are located in distinct niches in the subventricular zone (SVZ) of the lateral ventricle and the dentate gyrus of the hippocampus that provide molecular cues for cell proliferation and differentiation. Similar to rodents and non‐human primates, NSCs undergo postnatal neurogenesis in humans, but the exact age at which neurogenesis declines remains controversial (Boldrini et al., 2018; Ernst et al., 2014; Kempermann et al., 2018; Moreno‐Jimenez et al., 2019; Sorrells et al., 2018).

*In vivo*, the majority of NSCs reside in a state of quiescence (Codega et al., 2014). Quiescent NSCs (qNSCs) have exited the cell cycle but can be prompted by intrinsic or extrinsic cues to activate (aNSCs) and proliferate, before returning to quiescence or differentiating into neurons or glia. NSC activation is the first critical step in adult neurogenesis, and can be enhanced in response to damage (e.g., stroke) or environmental stimuli such as parabiosis (Parent et al., 2002; Villeda et al., 2011; Zhang et al., 2014). Evidence shows that decreased neurogenesis with age occurs due to reduced NSC activation, senescence of the NSC niche, and exhaustion of the qNSC pool (Daynac et al., 2016; Encinas et al., 2011; Enwere et al., 2004; Luo et al., 2006). However, the precise mechanisms that prompt qNSCs to re‐enter the cell cycle in the healthy mammalian brain are mostly unknown.

Recent studies have reported that qNSCs and aNSCs employ cell type‐specific mechanisms to support their functionality, including distinct metabolic states and differences in proteostasis (Beckervordersandforth et al., 2017; Knobloch et al., 2017; Leeman et al., 2018; Morrow et al., 2020). Transcriptional profiling of qNSCs and aNSCs revealed both shared and distinct signatures, indicating that during NSC activation, a transcriptional overhaul occurs at a subset of genes involved in cell proliferation, lipid metabolism, and proteostasis (Codega et al., 2014; Dulken et al., 2017; Leeman et al., 2018; Morizur et al., 2018). Similar changes have been observed using *in vitro* models of NSC quiescence and activation (Marqués‐Torrejón et al., 2021; Martynoga et al., 2013), which provide an opportunity for more in‐depth mechanistic studies. These findings raise the question of how distinct transcriptional states are established across the NSC lineage, and how specific transcriptional changes are regulated at the chromatin level to drive neurogenesis.

Here, we used the assay for chromatin accessibility (ATAC‐seq) to map genomic chromatin states in quiescent and activated NSCs with age. We observe a strong association between chromatin accessibility, gene expression, and binding of key transcriptional regulators. Interestingly, we observe that in quiescent, but not activated NSPCs, accessibility is reduced with age at genes supporting proliferation and metabolic functions, suggesting a chromatin‐level mechanism for decreased neurogenesis in the aged brain.

## 2 RESULTS

### 2.1 Quiescent and activated NSPCs harbor shared and distinct chromatin profiles

**FIGURE 1:**
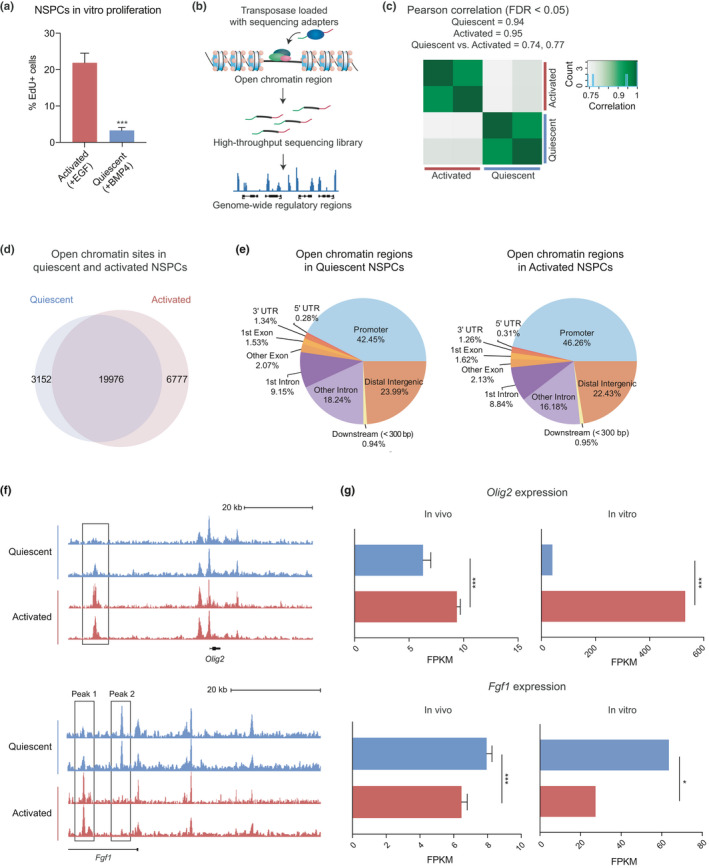
Quiescent and activated NSPCs have shared and differentially accessible chromatin regions. (a) EdU incorporation assay of activated and quiescent NSPCs (*n* = 3, ***p < 0.001, Student's t‐test, mean ±SD). (b) Schematic of the ATAC‐seq method. (c) Pearson correlation coefficient r between ATAC‐seq biological replicates (0.94 in quiescent, 0.95 in activated). (d) Shared and unique accessible chromatin sites in quiescent and activated NSPCs (FDR <0.05). (e) Genomic distribution of all open chromatin in quiescent and activated NSPCs. (f) UCSC genome browser shots of ATAC‐seq tracks at the *Olig2* (top) and *Fgf1* loci (bottom). (g) Differential expression of *Olig2* and *Fgf1* in quiescent and activated NSCs *in vivo*, and in NS5 cells *in vitro* (***FDR <0.001, *FDR <0.05)

To investigate how transcriptional changes underlying NSC activation are associated with chromatin accessibility, we performed ATAC‐seq (Buenrostro et al., 2013) on quiescent and activated primary mouse neural stem and progenitor cells (NSPCs; primary NSCs in culture contain a mixture of stem and progenitor cells (Pastrana et al., 2011)). We isolated NSPCs from young and old mouse brains and collected nuclei from early passage cells cultured in activated/growth conditions or quiescent conditions, as previously established (Leeman et al., 2018; Martynoga et al., 2013; Mira et al., 2010). Activated NSPCs were maintained in proliferation media containing EGF and FGF2, whereas quiescent NSPCs were cultured in BMP4 (Bone Morphogenetic Protein 4) and FGF2, which induces a rapid, reversible quiescent state in NSPCs (Marqués‐Torrejón et al., 2021; Martynoga et al., 2013; Mira et al., 2010). Cell cycle status under the two conditions was confirmed using a two‐hour EdU (5‐ethynyl‐2’‐deoxyuridine) incorporation assay (*p* < 0.001, Student's t‐test) (Figure 1a and S1A‐B). To demonstrate that quiescent NSPCs retain the potential for cell cycle re‐entry, we reactivated the BMP4‐treated cells by removing BMP4 and adding back EGF (Figure S1A,B). Consistent with previous research suggesting that aged NSCs show reduced activation (Leeman et al., 2018) we found that NSPCs from old brains showed a decreased proliferation rate in activated and reactivated states compared to young cells (Figure S1A). Lastly, we confirmed that NSPCs isolated from both young and old brains expressed the stem cell marker SOX2 regardless of their cell cycle status, and higher levels of NESTIN in actively dividing NSPCs, consistent with previous findings in the SVZ (Figure S1B, Codega et al., 2014).

Using this quiescence model system, we generated ATAC‐seq libraries in quiescent and activated NSPCs to map the genomic changes in chromatin accessibility in the two cellular states (Figure 1b). Initial analysis revealed that quiescent and activated NSPCs have over 43,000 accessible chromatin sites (Table S1). We found that ATAC‐seq signals between two biological replicates in each condition were highly correlated (r = 0.94 in quiescent and 0.95 in activated, Pearson correlation coefficient) (Figure 1c). Next, we used DiffBind to identify the shared and differential ATAC‐seq signals between quiescence and activation (Stark & Brown, 2011). Interestingly, we found that approximately 67% of the accessible sites (19,976 sites) were shared between the two conditions. These sites represent stably accessible chromatin regions that do not change significantly between the two cellular states, defining an accessibility signature of the neurogenic NSPCs (Figure 1d). Accessible sites in quiescent and activated NSPCs were similarly distributed across the genome (Figure 1e). Comparison of the NSPC ATAC‐seq signals to those from postnatal mouse lung, liver, intestine, kidney, and stomach tissues (ENCODE) (Sloan et al., 2016) showed that quiescent and activated NSPCs exhibit a distinct chromatin profile from other cell lineages (Figure S1C).

We next examined how chromatin accessibility is altered between quiescent and activated NSPCs at key genomic regions for NSC self‐renewal and proliferation, *Olig2* and *Fgf1* (Mateo et al., 2015; Sun et al., 2011). An upstream region near *Olig2* was more “Accessible in Activated,” or “AA” as indicated by ATAC‐seq signals (Figure 1f, top). In the *Fgf1* locus, we observed an AA site (Peak 1), and another site that was more “Accessible in Quiescent,” or “AQ” (Peak 2) (Figure 1f, bottom). Consistent with the changes in chromatin, *Olig2* and *Fgf1* are differentially expressed in quiescent and activated NSCs (Figure 1g) in two independent RNA‐seq datasets: 1) freshly isolated NSCs purified by fluorescence‐activated cell sorting (FACS) (Leeman et al., 2018) and 2) cultured NS5 cells (an embryonic stem cell derived neural progenitor line) (Martynoga et al., 2013). Furthermore, ChIP‐seq datasets for enhancer marks suggest that these dynamic peaks may have functional enhancer activity (Figure S1D). Globally, 9,929 sites had altered accessibility between quiescent and activated NSPCs, consisting of 3,152 AQ and 6,777 AA sites (FDR <0.05) (Figure 1d and Table S2). Together, these results reveal that quiescent and activated NSPCs exhibit similarities in global chromatin profiles, with quiescence and activation‐specific differences in approximately one third of the accessible chromatin.

### 2.2 Dynamic chromatin regions are associated with gene regulation in NSC activation

**FIGURE 2:**
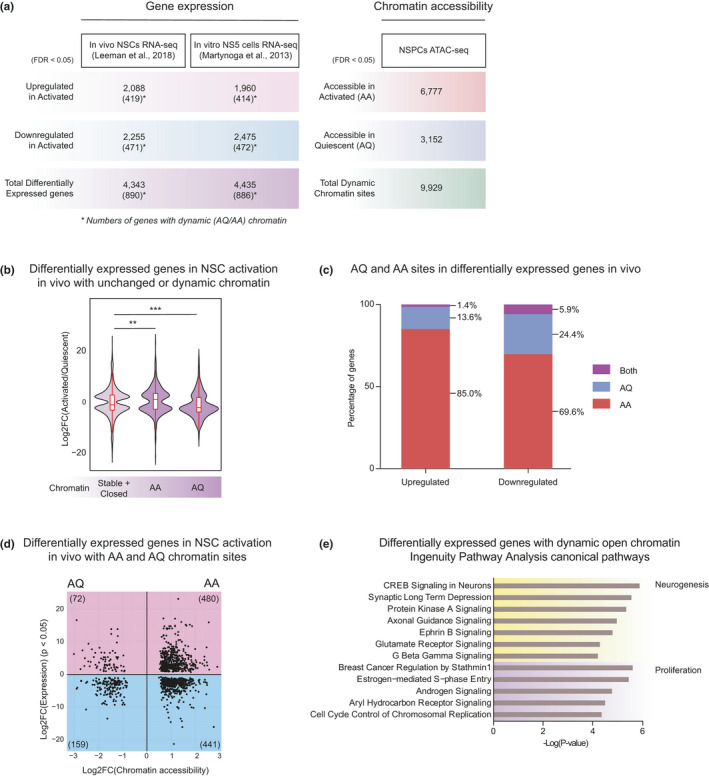
Dynamic chromatin regions are associated with differential expression of a neurogenesis gene network. (a) Summary of the differentially expressed genes in two independently generated RNA‐seq datasets and differentially accessible chromatin sites in ATAC‐seq (FDR <0.05). Numbers in parentheses indicate differentially expressed genes with dynamic chromatin sites. (b) Comparison of differential expression *in vivo* between genes with chromatin with no change, and with dynamic chromatin (***p < 0.001, **p < 0.01, Wilcoxon rank sum test). (c) Percentages of differentially expressed genes that contain dynamic chromatin sites. (d) Scatterplot showing fold change in chromatin accessibility versus fold change in associated gene expression (FDR <0.05). Each dot is a dynamic chromatin site associated with a differentially expressed gene, and the total number of sites in each quadrant is shown. (e) IPA analysis of genes differentially expressed *in vivo* and associated with dynamic chromatin (top 12 pathways shown)

To compare the transcriptomic changes between quiescent and activated NSPCs with chromatin‐level changes we observed, we first utilized the two RNA‐seq datasets described above (Figure 2a). We asked whether differentially expressed genes between quiescence and activation exhibit dynamic (AQ/AA) accessibility in the two cellular states. Of all genes differentially expressed upon NSC activation, approximately 20% were associated with chromatin changes (AA/AQ chromatin; *in vivo* 20.4% and *in vitro* 20.0%) (Figure 2a). Overall, we found that genes with opening chromatin were significantly more upregulated upon NSC activation than genes without chromatin changes (Figure 2b and S2A‐B). Among the upregulated genes with dynamic chromatin, most were associated with AA sites (85.0%; Figure 2c and S2A). In contrast, genes with AQ sites displayed greater downregulation and 24.4% of genes downregulated in aNSCs were associated with chromatin closing in the activated state (AQ sites; Figure 2b,c). These findings are consistent with a model in which increased chromatin accessibility at specific sites allows for recruitment of transcription factors to drive an aNSC‐specific gene expression program. Moreover, ATAC‐seq on reactivated NSPCs revealed that nearly all AA chromatin became accessible again upon re‐entry into the cell cycle, further supporting the importance of dynamic chromatin remodeling upon exit from quiescence (Figure S3). Notably, we observed that many genes that were downregulated upon NSC activation contain AA sites (69.6%; Figure 2c,d), suggesting that transcriptional repression is also associated with chromatin remodeling at these target genes. Finally, to probe the functional relevance of changes in chromatin accessibility, we tested whether dynamic chromatin sites were enriched for particular signaling pathways using Ingenuity Pathway Analysis (IPA) (Kramer et al., 2014) (Figure 2e). Interestingly, genes with dynamic chromatin were most highly enriched with neural identity, differentiation, and proliferation pathways.

In order to validate the above findings and identify the core transcriptional and epigenetic programs that define the quiescent and activated states and how they change with age, we performed RNA‐seq on quiescent and activated NSPCs from young and aged mice. We first compared the young transcriptional profiles with those from *in vivo* NSCs and *in vitro* NS5 cells (Figure S2D). Overall, we observed strong overlap between gene expression changes in qNSCs versus aNSCs in all systems (*in vivo* NSCs, *in vitro* NS5 cells, and NSPCs), although comparison of genes downregulated in aNSCs versus qNSCs *in vivo* and in our primary culture did not reach statistical significance. This may be due to additional quiescence‐promoting signals in the SVZ niche that are absent in the *in vitro* culture system, as the overlap between downregulated genes in BMP4‐treated NS5 cells and NSPCs was significant (Figure S2D). Furthermore, overlapping upregulated genes with NSC activation were enriched in cell cycle signaling pathways, while shared downregulated genes were enriched in G protein‐coupled receptor signaling pathways which are associated with quiescence (Codega et al., 2014) (Table S10). Together, these results show that while there are some differences in global gene expression profiles among *in vivo* NSCs, ES‐derived neural progenitor NS5 cells, and primary cultured NSPCs, expression levels of key genes involved in neurogenic cell identity and cell cycle progression are shared across systems.

### 2.3 Constitutively accessible sites are enriched for H3K4me3 and specific promoter elements

**FIGURE 3:**
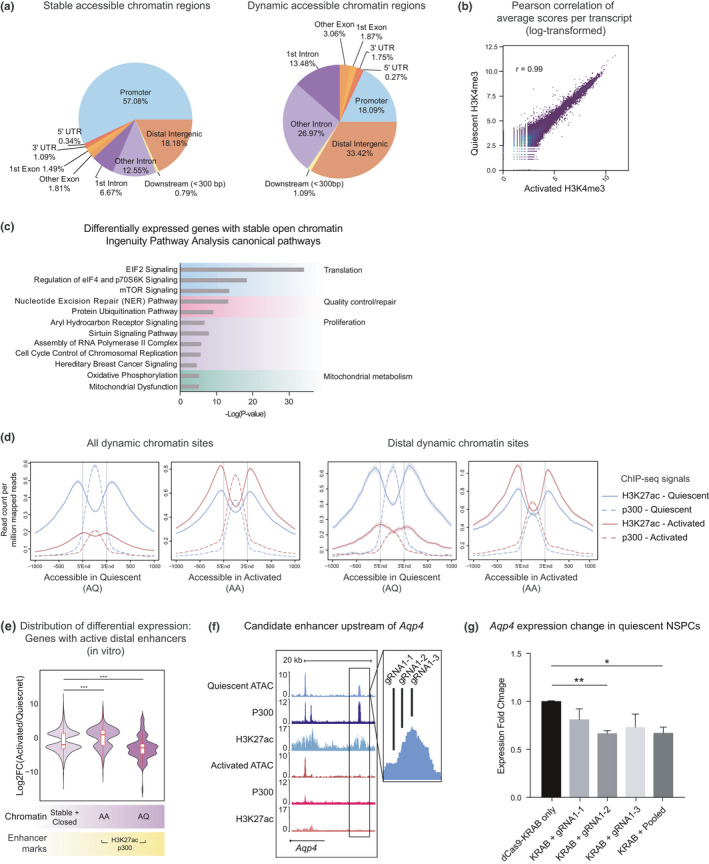
Distinct chromatin features are associated with constitutively accessible and dynamic chromatin in NSPCs. (a) Genomic distribution of stably open and dynamic chromatin regions in quiescent and activated NSPCs. (b) Pearson correlation of average H3K4me3 ChIP‐seq MACS enrichment scores per gene between quiescent and activated NSPCs (*r* = 0.99). (c) Top 12 IPA Canonical Pathways enriched in genesets that are differentially expressed between quiescent and activated NSCs *in vivo* and harbor only constitutively open chromatin. (d) Dynamic chromatin sites are enriched for features of active enhancers. H3K27ac and p300 ChIP‐seq signals in quiescent and activated NSPCs in all AQ/AA chromatin regions (left) and in distal AQ/AA chromatin (right). (e) Comparison of differential expression of genes with no change in chromatin and dynamic chromatin with active enhancer marks (***p < 0.001, Wilcoxon rank sum test). (f) UCSC genome browser shot of the putative *Aqp4* enhancer and gRNAs targeting the enhancer region. G, RT‐qPCR of *Aqp4* by dCas9‐KRAB with gRNAs targeting the enhancer region. Fold change is relative to the dCas9‐KRAB only control (*n* = 3 experiments, **p < 0.01, *p < 0.05, Student's *t*‐test, mean ±SD)

Our observation that many chromatin regions are readily accessible in both the quiescent and activated states raised the question of how these sites function mechanistically to support the NSC lineage. Stably open chromatin regions were associated with 12,779 genes (Table S3), 10,683 of which were exclusively associated with stable chromatin (no dynamic chromatin). Moreover, genes with only stable open chromatin were associated with upregulated expression in aNSCs (*p* = 9.78 × 10^−30^ *in vivo*, *p* = 7.63 × 10^−5^ *in vitro*, Wilcoxon rank sum test). Our finding that both dynamic and stable chromatin states could be linked to transcriptional activation suggests that transcriptional changes in NSC activation are supported by at least two distinct mechanisms. Consistent with this possibility, we observed that dynamic and stable chromatin sites had different distributions relative to transcription start sites (TSSs) (Figure 3a). Most stable chromatin regions were found in promoters (57.08% −1 kb/+1 kb from TSSs), whereas dynamic chromatin was most frequent in distal intergenic (33.42%) or intronic (40.45%) regions. Thus, the major differences in chromatin accessibility in quiescent versus activated NSPCs occur at sites away from promoters, possibly at gene‐specific regulatory elements. Moreover, our observation that many promoters are readily accessible in quiescent and activated NSPCs suggests that promoter accessibility may be critical for rapid toggling between quiescence and activation without chromatin remodeling. Consistently, the histone modification associated with TSSs, H3K4me3, was correlated with stable chromatin regions and highly correlated between the quiescent and activated NSPCs (*r* = 0.99, Pearson correlation coefficient) (Figure 3b and S4A, Table S4). Moreover, more than 70% of differentially expressed genes were marked by H3K4me3 in both states, suggesting that differentially expressed gene promoters are primed with H3K4me3. In contrast, the H3K4me3 signals in NSPCs were distinct from those of various adult and embryonic mouse tissues (Shen et al., 2012), indicating that the H3K4me3 profile is neurogenic lineage‐specific (Figure S4B).

Next, we performed enrichment analysis to identify the cellular pathways associated with stable chromatin. Interestingly, genes differentially expressed with NSC activation and exclusively harboring stable chromatin were enriched for canonical pathways regulating translation, proteostasis, metabolism, RNA polymerase II assembly, and proliferation (Figure 3c and Table S5), in contrast with high enrichment of neural identity and proliferation in dynamic chromatin (Figure 2f). To better understand how genes lacking dynamic chromatin undergo transcriptional regulation, we performed an enrichment analysis of core promoter elements in genes upregulated with NSC activation using ElemeNT (Sloutskin et al., 2015). Analysis of upregulated genes in the top quartile (FDR <1.44 × 10^−7^) revealed that they were enriched with the TATA box or human TCT elements (*p* = 1.14 × 10^−5^ and 1.14 × 10^−4^, hypergeometric analysis) (Table S6). Intriguingly, the upregulated genes with these promoters are closely associated in a “translation cluster” comprising ribosomal proteins, consistent with the enrichment of translation and proteostasis pathways in the stable open chromatin and with the enrichment of the TCT motif in promoters of ribosomal protein genes (Figure S4C,D) (Parry et al., 2010). Thus, specific promoter elements at constitutively accessible promoters are likely involved in fine tuning transcriptional activity to support changes in basal metabolic states as stem cells activate.

### 2.4 A subset of distal chromatin regions with changing accessibility during NSPC activation are active enhancers

The majority of the dynamic chromatin sites between quiescent and activated cells (73.87%) reside in distal regions (Figure 3a), suggesting that they may be functional enhancers. We analyzed markers of active enhancers (H3K27ac and p300 ChIP‐seq signal) from neural progenitors either actively dividing or induced to enter quiescence (Martynoga et al., 2013), and found a strong enrichment for active enhancer marks at dynamic chromatin sites, confirming that distal chromatin regions with dynamic accessibility harbor quiescent and activated neural stem lineage‐specific active enhancers (Figure 3d). Importantly, changes in accessibility at these active enhancers correlate with transcriptional regulation of their associated genes in NS5 cells (Figure 3e and S5A). We observed a number of differences between active enhancers in distal AA sites and *in vivo* gene expression, although it did not reach statistical significance (Figure S5B,C). This is likely because chromatin opening at active enhancers is associated with both up and downregulation of gene expression, consistent with our finding in global chromatin opening (Figure 2b). In distal AQ sites, active enhancers were significantly associated with decreased gene expression *in vivo* and *in vitro*.

We next asked whether targeting enhancers in dynamic chromatin was sufficient for gene regulation. We identified an upstream enhancer region (chr18:15421139–15421834) near *Aqp4*, which is expressed in quiescent NSCs and astrocytes and involved in NSC proliferation, migration, and differentiation (Kong et al., 2008) (Figure S5D). The putative enhancer region was accessible with active enhancer marks only in quiescent NSPCs (Figure 3f). We used dCas9‐KRAB (Klann et al., 2017) to repress the *Aqp4* enhancer in quiescent NSPCs (Figure S5E). We found that one of the three gRNAs targeting this region in dCas9‐KRAB‐expressing, quiescent NSPCs was sufficient to downregulate *Aqp4* expression (*p* = 0.0024, Student's *t*‐test) (Figure 3g). These results demonstrate that enhancers residing in dynamic chromatin function in gene regulation, and targeting a distal element can modulate gene expression.

Next, to identify the transcription factors functioning at enhancers in dynamic chromatin, we performed *in silico* motif analyses to identify enriched consensus sequences (Figure S6A). Interestingly, dynamic chromatin harbored distinct binding motifs for pro‐quiescence and proliferation factors, Nuclear Factor‐I (NFI) in AQ sites (*p* = 1 × 10^−113^) and ASCL1 (basic helix‐loop‐helix) in AA sites (*p* = 1 × 10^−372^) (Castro et al., 2011; Martynoga et al., 2013; Parras et al., 2004). We confirmed the binding of these factors using previously published ASCL1 and pan‐NFI ChIP‐seq datasets from activated and quiescent NSPCs (Martynoga et al., 2013; Webb et al., 2013) (Figure S6B,D). Interestingly, stably open chromatin was most highly enriched for CTCF motifs (*p* = 1 × 10^−1169^), and we confirmed CTCF binding with an available dataset (Beagan et al., 2017) (Figure S6E‐F). Altogether, our findings suggest that chromatin sites with dynamic accessibility between quiescent and activated cells harbor critical regulatory elements for NSC activation. Furthermore, CTCF occupancy at constitutively accessible sites represents chromatin boundaries that are present in open chromatin to stabilize these sites during NSPC activation.

### 2.5 Distinct transcriptional networks are associated with dynamic chromatin

**FIGURE 4:**
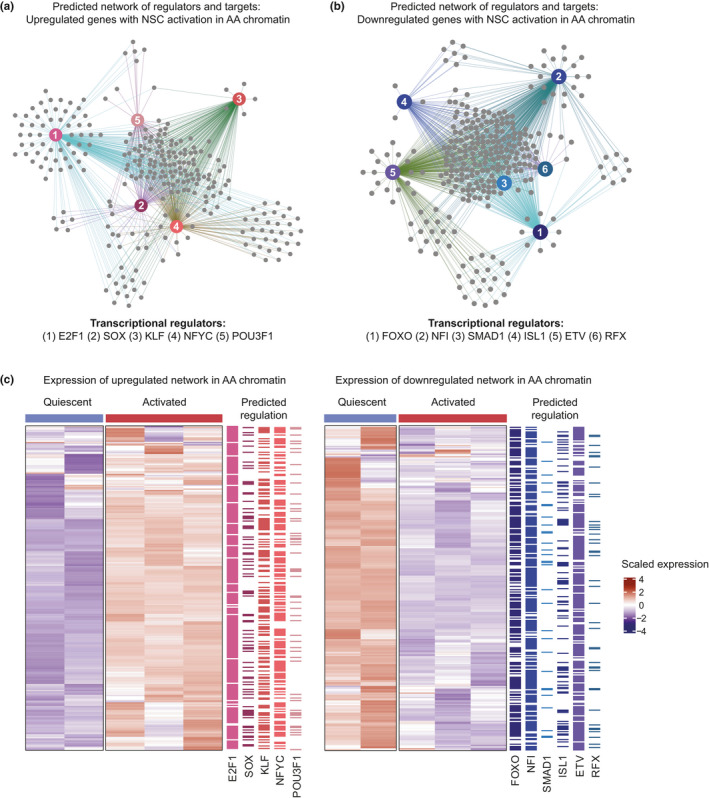
Dynamic chromatin is associated with specific transcription factor networks supporting activation and return to quiescence. (a‐b) Predicted regulators of upregulated (a) and downregulated (b) genes with AA chromatin. Gray nodes represent upregulated genes. Edges are colored according to predicted regulation by transcription factor nodes in red. (c) Heatmaps of up‐ and downregulated networks between quiescent and activated NSCs *in vivo* (FDR <0.05). Predicted regulation by each transcription factor is shown to the right

The unexpected association between AA chromatin and both up‐ and downregulation of gene expression (Figure 2d) suggests that there may be distinct transcription factors activating or repressing expression as chromatin regions gain accessibility in activated NSPCs. To examine this possibility, we performed a meta‐analysis of transcription factor consensus motifs to predict transcriptional regulators and target networks in AA chromatin (Figure 4a‐b and Table S8) (Heinz et al., 2010; Janky et al., 2014; Qin et al., 2020). 264 out of 362 upregulated and 286 out of 356 downregulated genes were integrated into the networks as targets of the candidate regulators. We found that AA‐associated up‐ and downregulated genes were predicted targets of distinct sets of transcription factors. Upregulated genes associated with AA chromatin were putative targets of E2F1, SOX, KLF, NFYC, and POU3F1, which have been implicated in stem cell proliferation and differentiation, pioneer factor function, and nucleosome eviction (Dodonova et al., 2020; Julian & Blais, 2015; Laub et al., 2005). In contrast, FOXO, NFI, SMAD1, ISL1, ETV, and RFX factors were predicted to bind genes downregulated with NSC activation. FOXO, NFI, and SMAD1 have known functions in maintaining NSC quiescence (Martynoga et al., 2013; Mira et al., 2010; Paik et al., 2009; Renault et al., 2009; Webb et al., 2013). Altogether, this meta‐analysis identified transcriptional hubs in opening chromatin associated with NSC activation, and suggests that distinct gene regulatory networks are part of the dynamic chromatin landscape that facilitates the return to quiescence after activation.

### 2.6 Quiescent NSPCs in the aged brain exhibit chromatin accessibility loss at select genes involved in metabolism

**FIGURE 5:**
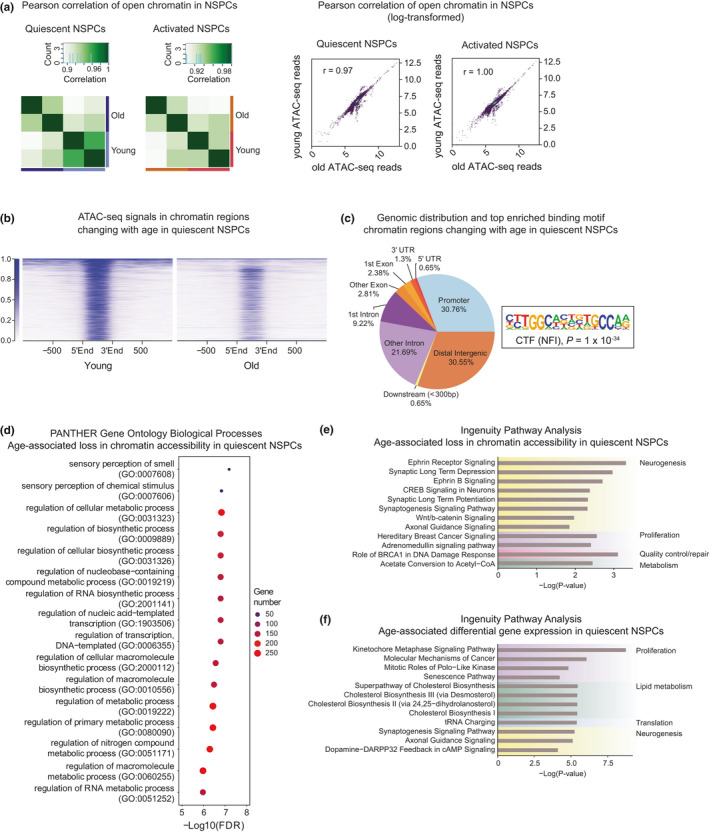
Quiescent NSPCs exhibit age‐associated accessibility changes in select chromatin regions involved in metabolism. (a) Pearson correlation and scatterplots of quiescent and activated NSPCs from young and old brains. (b) ATAC‐seq signals from young and old quiescent NSPCs in chromatin regions differentially accessible with age (FDR <0.05). (c) Genomic distribution of the differentially accessible regions and the top enriched motif at these sites. (d) PANTHER biological processes and (e) IPA pathways associated with differential accessibility in aged quiescent NSPCs (FDR <0.05, Benjamini–Hochberg correction). (f) Top IPA Canonical Pathways enriched in genes that change in expression in quiescent NSPCs with age

Consistent with the known decrease in neurogenesis with age (Audesse & Webb, 2020; Corenblum et al., 2016; Daynac et al., 2016), we observed that NSPCs isolated from aged mice had a reduced proliferation rate compared to young (Figure S1A,B). To elucidate the age‐associated chromatin changes in NSC quiescence and activation, we compared the young ATAC‐seq and RNA‐seq profiles to datasets collected in parallel from aged NSPCs isolated from 20–24 month‐old mice. We observed that quiescent NSPCs exhibited more age‐associated changes in chromatin accessibility than their activated counterparts (Figure 5a). In quiescent NSPCs, 1,388 chromatin sites were differentially accessible with age, while only 23 chromatin sites changed in activated NSPCs (Table S9). Notably, 98% of the sites with chromatin changes in quiescent NSPCs lost accessibility with age, and were highly enriched with the NFI binding motif (Figure 5b,c). Furthermore, many of these chromatin regions changing with age were located in distal intergenic regions and are binding sites of NFI factors (493 sites) (Martynoga et al., 2013) as well as FOXO3 (624 sites) (Webb et al., 2013), suggesting that age‐associated chromatin changes occur at pro‐quiescence gene regulatory regions (Figure 5c).

We next investigated whether the observed chromatin changes were linked to gene expression changes with age in NSPCs. We performed differential expression analysis on the RNA‐seq libraries described above from quiescent and activated NSPCs from young and old brains. To benchmark the dataset, we examined the expression levels of known quiescence‐ and proliferation‐associated marker genes in young and old NSPCs (Figure S7B,C), and found that NSPCs at both ages showed stem cell marker expression levels consistent with previous reports *in vivo* (Leeman et al., 2018; Morizur et al., 2018). We found that 2,207 genes were differentially expressed in quiescent NSPCs with age, and 491 genes in activated NSPCs (Table S10). Consistent with the ATAC‐seq data, age‐associated changes in gene expression were more abundant in quiescent NSPCs than activated NSPCs. Globally, our RNA‐seq data correlated with the *in vivo* NSCs datasets, with gene expression profiles of quiescent NSPCs correlated with qNSCs *in vivo*, and activated NSPCs better correlated with aNSCs *in vivo* (Figure S7A,B). Taken together, these results demonstrate that the chromatin and gene expression data from quiescent and activated NSPCs utilized in this study sufficiently represent the transcriptional landscape of NSCs *in vivo*, and that qNSCs undergo more significant transcriptional and epigenetic reprogramming with age than aNSCs.

To identify the biological processes associated with chromatin regions losing accessibility with age in quiescence, we performed enriched Gene Ontology analyses (PANTHER and IPA) (Figure 5d‐e). Interestingly, regulation of metabolism and transcription were among the most highly enriched biological processes, consistent with the notion that chromatin‐level changes in quiescent NSPCs affect metabolic functions with age (Figure 5d). Moreover, top pathways enriched in the chromatin regions losing accessibility with age were Ephrin signaling, DNA damage response, as well as other neurogenic pathways (Laussu et al., 2014), suggesting that regions that regulate NSC activation are becoming less accessible with age (Figure 5e). Supporting these results, differentially expressed genes in young and old quiescent NSPCs were enriched in signaling pathways involved in cell cycle regulation and lipid metabolism (Figure 5f). Moreover, Reactome pathway analysis also showed an enrichment of metabolism‐associated pathways in the chromatin regions losing accessibility with age (Figure S8). This integrated analysis demonstrates that with age, quiescent NSPCs undergo alterations of DNA accessibility in key regions that support quiescence‐specific functions, and these changes are accompanied by genome‐wide disruptions in cell cycle and metabolic pathways.

## 3 DISCUSSION

**FIGURE 6:**
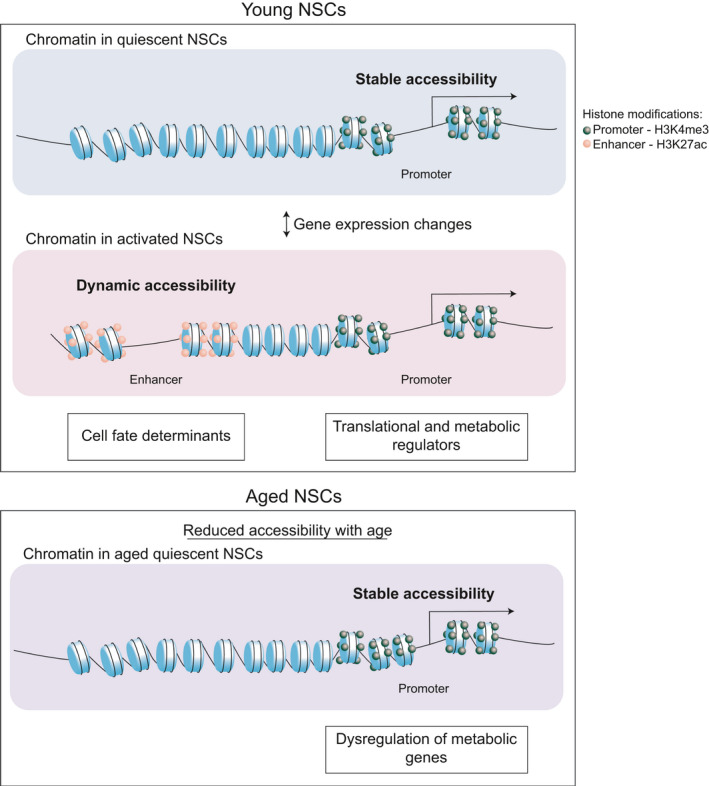
Model. NSC activation in the healthy brain is associated with transcriptional changes supported by stable and dynamic chromatin states. Aging is accompanied by dysregulation of these processes at metabolic genes in qNSCs. Only aged qNSCs are highlighted as the majority of age‐associated changes are observed in quiescent cells

We used ATAC‐seq to map the chromatin accessibility landscape of primary NSPCs in states of quiescence versus proliferation to uncover the chromatin‐level mechanisms supporting the first stage of mammalian neurogenesis, and define how they change with age (Figure 6). The *in vitro* quiescence system utilized in this study has been shown to be a reliable tool to study the transcriptional profiles of NSCs (Blomfield et al., 2019; Martynoga et al., 2013; Urbán et al., 2019), and we further benchmark this system here using RNA‐seq. Moreover, recent work characterizing this *in vitro* system has demonstrated that treatment of cultured NSCs with BMP4 and FGF results in a “primed” quiescent state, where NSCs readily exit quiescence and engage in neurogenesis when transplanted in the SVZ (Marqués‐Torrejón et al., 2021).

We find that, during aging, the majority of chromatin accessibility changes occur at metabolic genes in quiescent NSCs, suggesting an epigenetic mechanism underlying stem cell aging in the brain. Furthermore, RNA‐seq profiles of young and aged quiescent NSPCs revealed a large number of previously unknown genes that are differentially expressed in quiescence and aging. This dataset will serve as a valuable resource to the field of stem cell aging and adult neurogenesis, giving us new mechanistic insight into the transcriptional dysregulation that occurs with NSC aging. We also note that although our study uncovered few changes to glial gene networks with age, this may be due to the limited numbers of glia‐associated ontologies in available databases compared to ontologies involved in neurogenesis and neuronal maturation. These pathways may be changed in the model system used here, as BMP signaling has been shown to promote gliogenesis (Bonaguidi et al., 2005).

The ability of NSCs to exit and re‐enter quiescence is critical for maintenance of the stem cell pool in the adult and aged brain. NSC quiescence is a tightly regulated state characterized by a transcriptional program enriched for lipid metabolism, glycolysis, and specific cell signaling pathways. This transcriptional program is dramatically altered as NSCs activate, upregulating genes involved in oxidative metabolism, cell proliferation, and protein translation (Audesse & Webb, 2020; Llorens‐Bobadilla et al., 2015; Morizur et al., 2018; Shin et al., 2015). Moreover, with NSC activation, aggregates of damaged proteins and molecular cargo that accumulate in qNSCs are cleared. Recent work has shown that with age, proteostasis, aggregate clearance and metabolic pathways are altered in qNSCs, resulting in impaired activation (Leeman et al., 2018; Morrow et al., 2020; Zhao et al., 2016). Consistent with these studies, we observed that chromatin alterations and gene expression changes with age are more abundant in quiescent than activated NSCs. The observed overall loss of accessibility is consistent with work reporting reduced accessibility at promoters and enhancers in the immune system with age (Ucar et al., 2017). Our functional analysis linked decreased accessibility to metabolic functions and specific transcriptional regulators (FOXO3 and NFI), suggesting that these chromatin‐level changes contribute to the metabolic dysfunction in aged NSCs through disruption of the pro‐quiescence gene networks. FOXO3 has been implicated in maintaining healthy neurogenesis in the adult and aging brain (Audesse et al., 2019; Paik et al., 2009; Renault et al., 2009; Schäffner et al., 2018; Webb et al., 2013). Our discovery that many sites that change with age are occupied by FOXO3 suggests that either changes in FOXO3 activity may be responsible for the observed chromatin changes, or that epigenetic alterations prevent FOXO3 from occupying these sites. In future work, it will be important to relate the changes in the accessible chromatin landscape to specific metabolic changes with age, and to identify the precise relationship between epigenetic states, transcriptional networks and cellular metabolism in aging qNSCs.

## 4 EXPERIMENTAL PROCEDURES

### 4.1 Mouse NSPC cultures

Young (postnatal day 5) and aged (20–24 month) mouse NSPCs were isolated as previously described (Renault et al., 2009). Cells were cultured in high growth factor signaling conditions: Neurobasal A (ThermoFisher) medium with penicillin/streptomycin/glutamine (ThermoFisher), 2% B27 (ThermoFisher), and 20 ng/ml each of FGF2 (PeproTech) and EGF (PeproTech). To induce quiescence, 50,000 cells were first seeded in poly‐D‐lysine coated (Sigma) plates with high growth factor signaling medium, and after 24 h, fresh media without EGF and with 25 ng/ml recombinant mouse BMP4 (R&D Systems) was added. For reactivated NSPCs, cells were kept in BMP4‐containing Neurobasal A medium for 4 days, then switched to high growth factor signaling conditions for 9–12 days. Proliferation of the reactivated NSPCs was confirmed by 10 µM EdU (Sigma) incorporation for 2 h and detection (Click‐iT EdU Alexa Fluor 488 Imaging Kit, Invitrogen).

### 4.2 Immunocytochemistry

NSPCs were plated at 5 × 10^4^ cells/ml on poly‐D‐lysine treated coverslips. Cells were fixed with 4% paraformaldehyde for 10 min, blocked for 1 h with 5% goat serum/0.1% BSA, followed by incubation for 2 h at room temperature with primary antibody (SOX2 [1:200, EMD Millipore AB5603], NESTIN [1:200, BD Pharmingen #556309]). After washing five times with PBS/0.05% Tween‐20, coverslips were incubated with the appropriate secondary antibody for 1 h at room temperature (Molecular Probes Alexa Fluor, goat anti‐rabbit 488, goat anti‐mouse 546). Cells were imaged with a Zeiss Axiovert 200 M Fluorescence microscope.

### 4.3 ATAC‐seq

ATAC‐seq libraries were generated from early passage (2–4) NSPCs cultured in activated or quiescent conditions, with two biologically independent replicates per condition. Library preparations and quality analyses were performed as described (Buenrostro et al., 2013). Briefly, for activated NSPCs, 50,000 cells were seeded in Poly‐D‐Lysine coated plates in high growth factor signaling conditions as described above, and collected with Trypsin‐EDTA (ThermoFisher) after 24 h. For quiescent NSPCs, cells were switched to quiescence medium 24 h after plating and collected after 72 h. NSPCs were subjected to tagmentation reactions with 2.5 µl Tn5 Transposase (Illumina), purified with Qiagen MinElute PCR purification kit (Qiagen) and PCR‐amplified with 8–9 cycles. Due to age‐associated differences in NSPC proliferation rates, replicates were collected over a 12 day window and all transpositions were performed with the same batch of Tn5‐transposase. Quality of ATAC‐seq libraries was confirmed with Bioanalyzer (Agilent) prior to sequencing.

### 4.4 Processing of ATAC‐seq data

ATAC‐seq libraries were sequenced to a depth of approximately 40 million unique, high quality mapped reads per sample. 2 × 100 base pair paired‐end reads were trimmed with TrimGalore! (Version 0.4.0, Babraham Bioinformatics), and aligned to the most recent *Mus musculus* genome (mm10) using Bowtie2 (Version 2.2.5) (Langmead et al., 2009). Duplicates were marked with Picard (Version 1.88) and removed with SAMtools (Version 1.3.1). Peak calling was performed after ATAC‐seq‐specific quality control steps (Daugherty et al., 2017) using MACS (Version 2.1.1) (Zhang et al., 2008) with the FDR threshold 0.05. Peaks were assigned to genes using GREAT (Version 3.0.0) (McLean et al., 2010), limiting the peak‐calling window to −1 kb/+1 kb around TSSs for proximal gene assignments, and −25 kb/+10 kb around TSSs for distal assignments. Fastq files for ATAC‐seq datasets from mouse tissues, were downloaded from the ENCODE portal (Sloan et al., 2016) with the following identifiers: ENCSR609OHJ, ENCSR102NGD, ENCSR597BGP, ENCSR389CLN, ENCSR079GOY.

### 4.5 Differential accessibility analysis

The DiffBind package in R was used (Version 3.3.1) (Stark & Brown, 2011) to identify shared and unique ATAC‐seq peaks across quiescent and activated replicates. DiffBind was used to obtain Pearson's r correlation values between biological replicates, the corresponding heatmap, and differential accessibility analysis. Briefly, a unique “consensus peakset” was obtained by merging all overlapping peaks, and using the sequence reads (BAM) files to normalize numbers of reads for each sample at every accessible site. The “dba.report” function was used to identify differentially accessible intervals between conditions. For comparison of ATAC‐seq signals from young and old NSPCs, “block =DBA_REPLICATE” option was used to count sequencing reads across libraries, and “method =DBA_DESEQ2_BLOCK” was used for differential accessibility analysis to model batch effects from library preparation steps. ATAC‐seq signal enrichment in quiescent NSPCs from the two age groups in differentially accessible chromatin regions was plotted using NGS plot (Shen et al., 2014). Correlation plots for ATAC‐seq signals and Pearson correlation coefficient r were generated by the “plotCorrelation” function in deepTools (Version 3.4.3) (Ramirez et al., 2016).

### 4.6 Genomic distribution of ATAC‐seq signals

To determine the distribution of open chromatin regions from ATAC‐seq, the ChIPseeker package (Version 3.7) in R was used (Yu et al., 2015). BED files for quiescent and activated ATAC‐seq replicates were merged, and promoter regions were defined as −1kb/+1kb around TSSs. Peaks were annotated using “annotatePeak()” command, and pie charts were generated using “plotAnnoPie()”.

### 4.7 Chromatin immunoprecipitation

H3K4me3 ChIP‐seq libraries were generated from 1 × 10^7^ NSPCs. Chromatin was crosslinked with 1% formaldehyde for 10 min, followed by quenching with 0.125 M glycine for 5 minutes. Cells were washed with PBS pH 7.4, incubated in SDS lysis buffer (50 mM Tris‐HCl pH 7.5, 10 µM EDTA, 1% SDS) for 15 min on ice, and harvested by scraping. Nuclei were pelleted and resuspended in RIPA buffer (1% IGEPAL CA‐630, 0.5% sodium deoxycholate, 1% SDS in PBS pH 7.4), and chromatin was sheared with a Covaris S220 Focused‐ultrasonicator, at peak power 140 W, duty factor 10% and 200 Cycles/burst at 4°C. 5 μg H3K4me3 antibody (Abcam ab8580) coupled to anti‐Rabbit Dyna beads IgG (Invitrogen) was used per ChIP. Beads were pelleted and washed 1X with low salt, 2X with high salt, 3X with LiCl wash buffers (Millipore Sigma), and 2X with TE buffer (10 mM Tris‐HCl, 1 mM EDTA). DNA was eluted using elution buffer (4% SDS, 0.1 M NaHCO_3_) at 65°C for 2 h. Beads were removed, and crosslinks reversed overnight at 65°C. DNA isolation was performed using 25:24:1 phenol:chloroform:isoamyl alcohol, ethanol precipitation of DNA for 1 h at −20°C, and resuspension in 100 μl water. Libraries were generated by Genewiz (2 × 150 bp paired‐end sequencing; Illumina HiSeq).

### 4.8 Processing of ChIP‐Seq datasets

Raw sequencing reads (fastq files) were processed as previously described (Webb et al., 2016) with minor modifications for alignment to mm10. For publicly available datasets, fastq files were downloaded from ArrayExpress and Gene Expression Omnibus repositories. Briefly, after mapping with Bowtie2, duplicate reads were marked with Picard and removed with SAMtools. MACS was used to call peaks using the following commands: “macs2 ‐t ChIP.bam ‐c Input.bam ‐f BAM ‐g mm ‐B ‐q 0.001 ‐‐nomodel ‐‐extsize 150 ‐‐keepdup all”. For the CTCF ChIP‐seq dataset, q‐value cutoff of 1E‐8 was used. For the H3K4me3 and H3K27ac ChIP‐Seq datasets, an additional parameter of “‐‐broad” was used with “‐q 0.05 ‐‐broad‐cutoff 0.01”. Correlation plots for H3K4me3 ChIP‐seq signals were generated by deepTools “plotCorrelation” function. Peaks were assigned to genes using GREAT, with a window to −2 kb/+2 kb around TSSs. H3K27ac and p300 binding enrichment in dynamic chromatin was performed using NGS plot. ChIP‐seq datasets: E‐MTAB‐1423 (H3K27ac, p300 quiescent and activated NS5 cells, NFI quiescent NS5 cells), GSE48336 (ASCL1), GSE29184 (H3K4me3 in adult and embryonic mouse tissues), GSE85185 (CTCF from postnatal day 1 NSPCs).

### 4.9 RNA‐seq datasets

Generation and analysis of RNA‐seq datasets were described previously (Sequence Read Archive accession number SRP075993 for *in vivo* dataset) (Leeman et al., 2018; Martynoga et al., 2013). For this study, published normalized expression and fold change values were used.

### 4.10 RNA‐seq library preparation

Total RNA from quiescent and activated NSPCs was isolated from 2 × 10^5^ cells with the RNeasy mini kit (Qiagen). Samples were collected in triplicate for each age group and condition. PolyA libraries were generated using the NEB Next Ultra II library kit and sequenced by Genewiz.

### 4.11 RNA‐seq data processing

Transcripts were processed as previously described in the “new Tuxedo protocol” (Pertea et al., 2016). Briefly, Illumina adapters were removed using TrimGalore! (Version 0.4.0, Babraham Bioinformatics), and aligned to mm10 using Hisat2 (version 2.1.0) (Kim et al., 2015). Aligned reads were assembled using StringTie (version 1.3.4d) (Pertea et al., 2015), and expression level and transcripts were estimated. Differential expression was determined using DEseq2 (Love et al., 2014), FDR <0.05.

### 4.12 Violin plots and statistical analysis of differential expression

Violin plots were created in R using “ggplot2,” with the “geom_violin,” and “geom_boxplot” functions. Statistical analysis of the gene expression differences was performed using Wilcoxon rank sum test with continuity correction.

### 4.13 Venn diagrams and statistical analysis of overlaps

Venn diagrams were created in R using the “venneuler” package. Statistical analysis was performed using Fisher's exact test and all genes (with and without differential expression) (Martynoga et al., 2013) were used for the “background dataset.”

### 4.14 Lentiviral constructs and infection

gRNAs targeting a putative enhancer region upstream of *Aqp4* (chr18:15421139–15421834) were generated using GT‐Scan (O'Brien & Bailey, 2014) and selected based on minimal number of off‐targets. gRNAs (Table S7) were cloned into pLV‐U6‐gRNA‐UbC‐DsRed‐P2A‐Bsr (Addgene #83919) by Gibson assembly (Klann et al., 2017; Shalem et al., 2014). pLV‐dCas9‐KRAB‐PGK‐HygR (Addgene #83890) was described previously (Klann et al., 2017). Lentiviruses were produced in HEK293T cells using polyethylenimine (Polysciences) transfection with the packing plasmids PMDL, VSV‐G, and RSV (Durocher et al., 2002). After 24 h, transfection media was replaced with NSPC growth media and collected 24 hours later. To generate dCas9‐KRAB‐expressing cells, NSPCs were plated on poly‐D‐lysine at 50,000 cells/cm^2^ and infected with 1:1 ratio of growth media to viral supernatant. Virus was replaced with growth media after 24 hours. 3 days after infection, cells were selected with 700 µg/ml Hygromycin (Fisher Scientific) for 6 days. dCas9‐KRAB‐expressing NSPCs were induced into quiescence as described above, and simultaneously infected for 24 h with 1:1 ratio of growth media to gRNA lentiviral supernatant. RNA was collected 7 days after infection.

### 4.15 RT‐qPCR

Total RNA was isolated using the QIAGEN RNeasy kit with on column DNase digestion (QIAGEN). 250–500 ng RNA was used for reverse transcription, which was performed using the High Capacity cDNA Reverse Transcription Kit (Applied Biosystems). qPCRs were performed using Powerup SybrGreen Master Mix (Invitrogen) and run on an Applied Biosystems ViiA 7 Real‐Time PCR System. See Supplemental Table S7 for primer sequences used.

### 4.16 Motif analysis

Motif analysis was performed using the Homer findGenomeMotifs.pl tool (Heinz et al., 2010). Default settings (200 bp window) were used in all analyses, and *P*‐values from the Homer Known Motif Enrichment Results are reported. In cases where a motif appeared multiple times, only the top (smallest) *P*‐value is reported.

### 4.17 ElemeNT analysis

Core promoter analysis was performed using ElemeNT command line version 13 (Sloutskin et al., 2015). Constitutively accessible promoters of upregulated genes in NSC activation *in vivo* were divided into quartiles by rank (FDR). To detect element motifs, −50/+50 bp window around stably open TSS was analyzed. For each element, the positions from the input start position and corresponding PWM scores were reported. For enrichment analysis, −50/+50 bp window around TSS for all constitutively accessible gene promoters was used as background. Enrichment significance was calculated using the hypergeometric distribution and adjusted by Bonferroni correction. Only genes with element motifs in biologically relevant positions (−30/‐31 to −23/‐24 from TSS for the TATA box element, −1 to +6 from the TSS for the TCT motif, with −10/+10 bp allowance around expected positions) were highlighted.

### 4.18 Functional annotation

Functional annotation of genes was performed using Ingenuity Pathway Analysis (Kramer et al., 2014). PANTHER Classification System 16.0 was used for Gene Ontology biological process analysis (Mi et al., 2020), and *p*‐values were calculated by the binomial statistic, with Benjamini–Hochberg correction (Benjamini & Hochberg, 1995). Enriched biological processes and pathways were plotted in R using “ggplot2.”

### 4.19 Meta‐analysis of motif and transcription factor enrichment and network prediction

Unique motifs in genes upregulated and downregulated with *in vivo* NSC activation were identified using sequential analysis with Homer, LISA (epigenetic Landscape In Silico deletion analysis) (Qin et al., 2020), and iRegulon (Janky et al., 2014). Cytoscape (Shannon et al., 2003) was used to connect the transcription factors to their target genes, displaying regulation as interactions and genes as nodes. Differentially expressed genes that were not predicted to be regulated were excluded from network displays. Note that for the downregulated network, iRegulon's predicted transcription factor was FOXN, a member of the Forkhead transcription factor family. We verified that 87% of the predicted targets (39 out of 45 genes) were binding targets of FOXO3 in NSPCs (Webb et al., 2013).

## CONFLICT OF INTEREST

A.E.W. is a scientific co‐founder of Bolden Therapeutics, a company founded to develop treatments to replenish neurons in the adult brain.

## AUTHOR CONTRIBUTIONS

S.M‐L. and A.E.W. conceived and designed the study. S.M‐L. and S.D. harvested primary mouse NSPCs for experiments. S.M‐L. performed ATAC‐seq and ChIP‐seq with the supervision of A.E.W. S.M‐L. performed processing and analysis of all datasets. A.K.B. performed immunocytochemistry experiments. M.Y. set up the dCas9‐KRAB system. All remaining experiments were done by S.M‐L. A.S. and T.J‐G provided guidance on core promoter analysis, as well as helpful comments on the manuscript. S.M‐L and A.E.W. prepared the manuscript.

## Supporting information

Fig S1‐8

Table S1

Table S2

Table S3

Table S4

Table S5

Table S6

Table S7

Table S8

Table S9

Table S10

Supplementary Material

Supplementary Material

## Data Availability

Datasets are available through Gene Expression Omnibus accession number GSE161578. The code will be released at: https://www.github.com/Webb‐Laboratory/. All other resources used for this study are available through the corresponding author.
