# Supplementary material for: Changing and stable chromatin accessibility supports transcriptional overhaul during neural stem cell activation and is altered with age
Source: Aging Cell. 2021 Oct 23;20(11):e13499. doi: 10.1111/acel.13499 (PMC8590101; doi:10.1111/acel.13499)
Supplement: Supplementary file 1 — Fig S1‐8 [file ACEL-20-e13499-s005.pdf]

Fig. S1

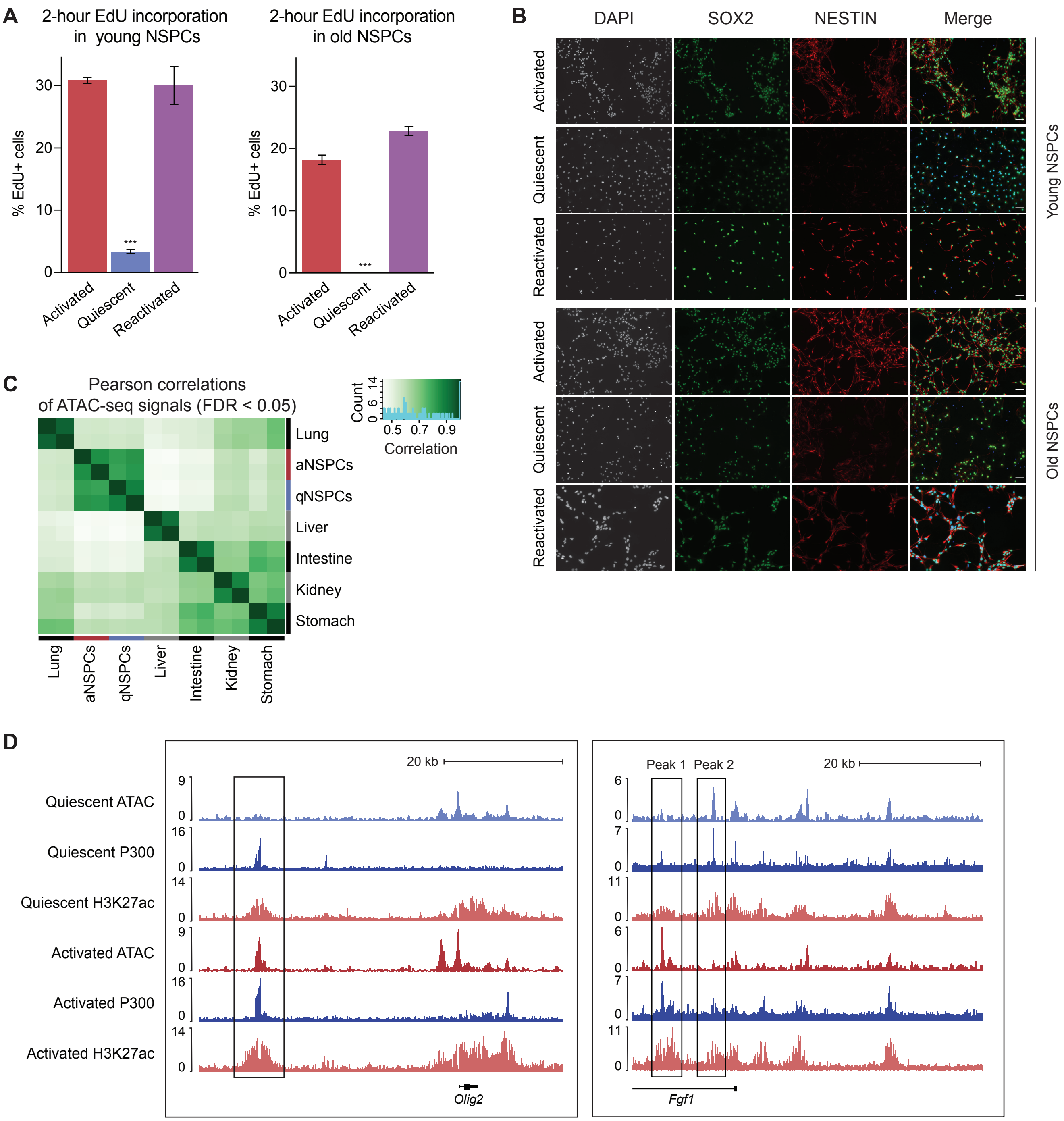
A
2-hour EdU incorporation in young NSPCs
2-hour EdU incorporation in old NSPCs
% EdU+ cells
0
10
20
30
***
Activated
Quiescent
Reactivated
B
DAPI
SOX2
NESTIN
Merge
Activated
Quiescent
Reactivated
Young NSPCs
Old NSPCs
C
Pearson correlations of ATAC-seq signals (FDR < 0.05)
Count
0 6 14
0.5 0.7 0.9
Correlation
Lung
aNSPCs
qNSPCs
Liver
Intestine
Kidney
Stomach
D
Quiescent ATAC
Quiescent P300
Quiescent H3K27ac
Activated ATAC
Activated P300
Activated H3K27ac
20 kb
Olig2
Peak 1
Peak 2
Fgf1

Fig. S2

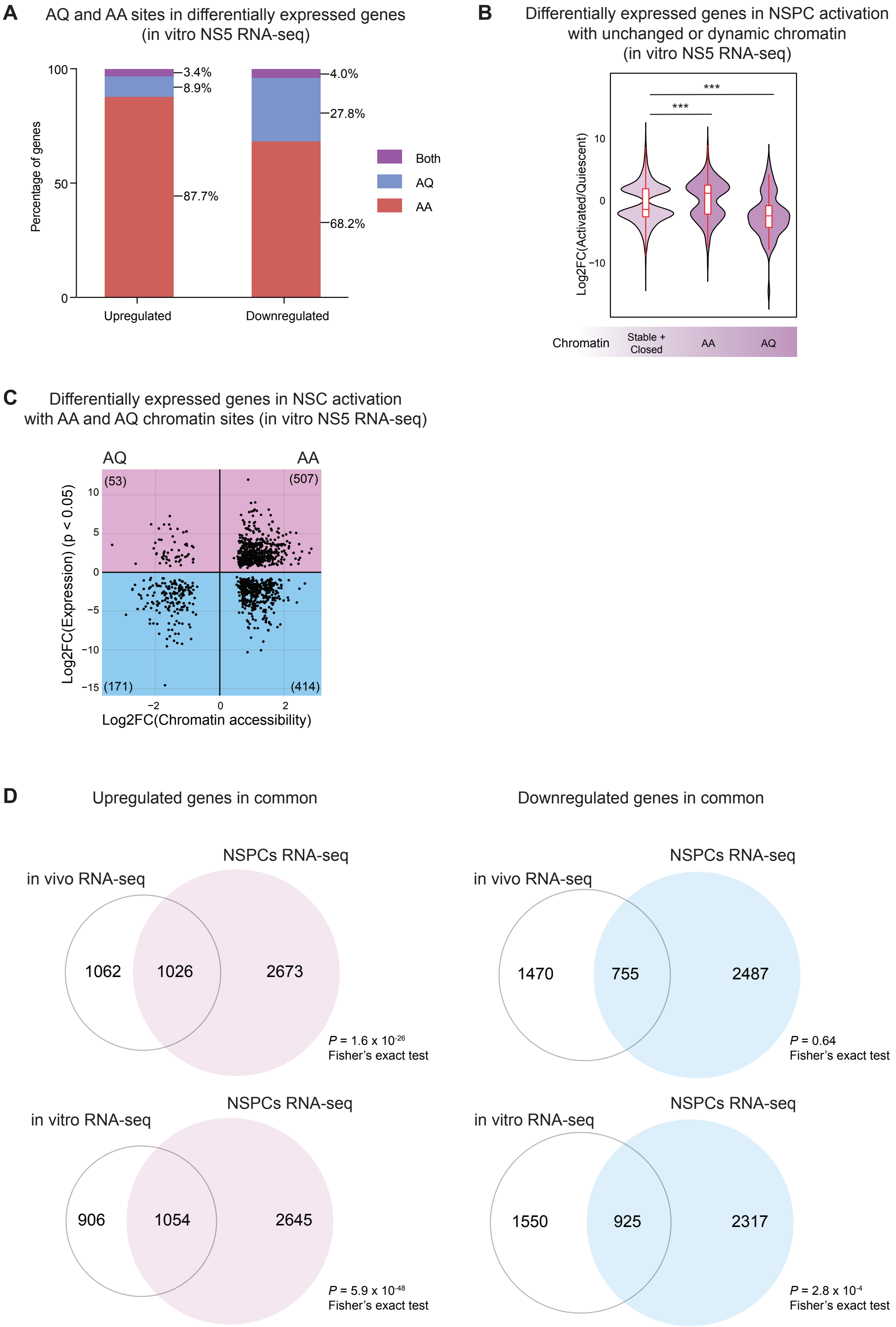
A
AQ and AA sites in differentially expressed genes
(in vitro NS5 RNA-seq)
100
50
0
Percentage of genes
3.4%
8.9%
87.7%
4.0%
27.8%
68.2%
Upregulated
Downregulated
Both
AQ
AA
B
Differentially expressed genes in NSPC activation
with unchanged or dynamic chromatin
(in vitro NS5 RNA-seq)
***
***
10
0
−10
Log2FC(Activated/Quiescent)
Chromatin
Stable +
Closed
AA
AQ
C
Differentially expressed genes in NSC activation
with AA and AQ chromatin sites (in vitro NS5 RNA-seq)
AQ
AA
(53)
(507)
(171)
(414)
10
5
0
−5
−10
−15
Log2FC(Expression) (p < 0.05)
−2
0
2
Log2FC(Chromatin accessibility)
D
Upregulated genes in common
Downregulated genes in common
in vivo RNA-seq
NSPCs RNA-seq
1062
1026
2673
P = 1.6 x 10-26
Fisher's exact test
in vivo RNA-seq
NSPCs RNA-seq
1470
755
2487
P = 0.64
Fisher's exact test
in vitro RNA-seq
NSPCs RNA-seq
906
1054
2645
P = 5.9 x 10-48
Fisher's exact test
in vitro RNA-seq
NSPCs RNA-seq
1550
925
2317
P = 2.8 x 10-4
Fisher's exact test

Fig. S3

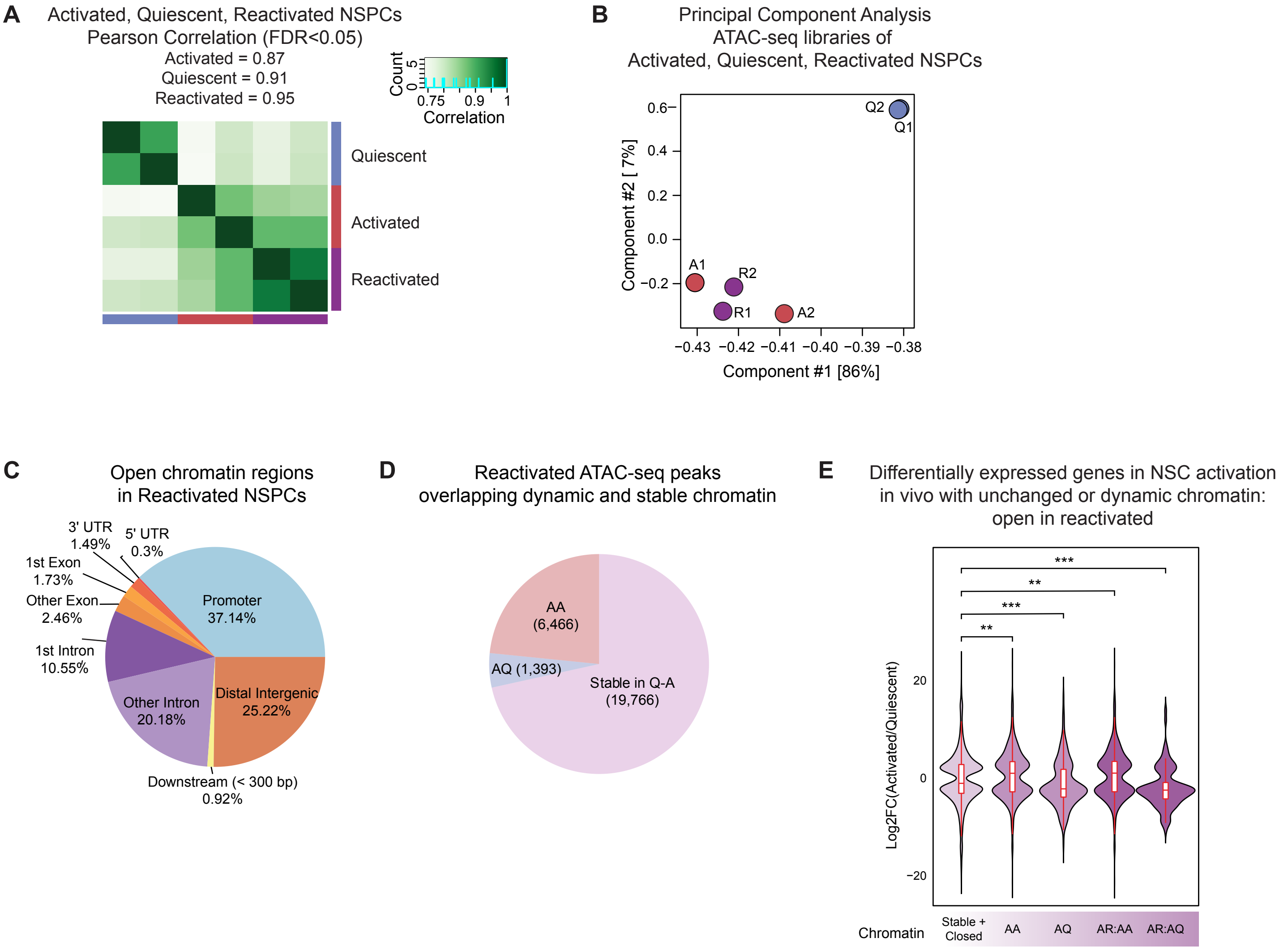
A
Activated, Quiescent, Reactivated NSPCs
Pearson Correlation (FDR<0.05)
Activated = 0.87
Quiescent = 0.91
Reactivated = 0.95
Count
0 5
0.75 0.9 1
Correlation
Quiescent
Activated
Reactivated
B
Principal Component Analysis
ATAC-seq libraries of
Activated, Quiescent, Reactivated NSPCs
Component #2 [7%]
0.6
0.4
0.2
0.0
−0.2
Q2
Q1
A1
R2
R1
A2
−0.43 −0.42 −0.41 −0.40 −0.39 −0.38
Component #1 [86%]
C
Open chromatin regions
in Reactivated NSPCs
3' UTR 1.49%
5' UTR 0.3%
1st Exon 1.73%
Other Exon 2.46%
1st Intron 10.55%
Promoter 37.14%
Distal Intergenic 25.22%
Other Intron 20.18%
Downstream (< 300 bp) 0.92%
D
Reactivated ATAC-seq peaks
overlapping dynamic and stable chromatin
AA (6,466)
AQ (1,393)
Stable in Q-A (19,766)
E
Differentially expressed genes in NSC activation
in vivo with unchanged or dynamic chromatin:
open in reactivated
***
**
***
**
Log2FC(Activated/Quiescent)
20
0
−20
Chromatin
Stable + Closed
AA
AQ
AR:AA
AR:AQ

Fig. S4

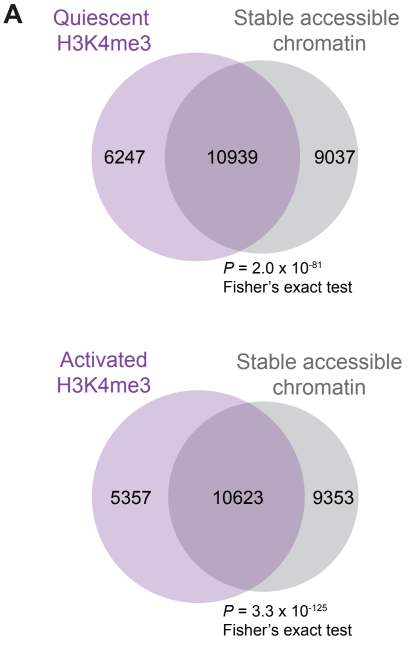
A
Quiescent H3K4me3
Stable accessible chromatin
6247
10939
9037
P = 2.0 x 10^{-81}
Fisher's exact test
Activated H3K4me3
Stable accessible chromatin
5357
10623
9353
P = 3.3 x 10^{-125}
Fisher's exact test

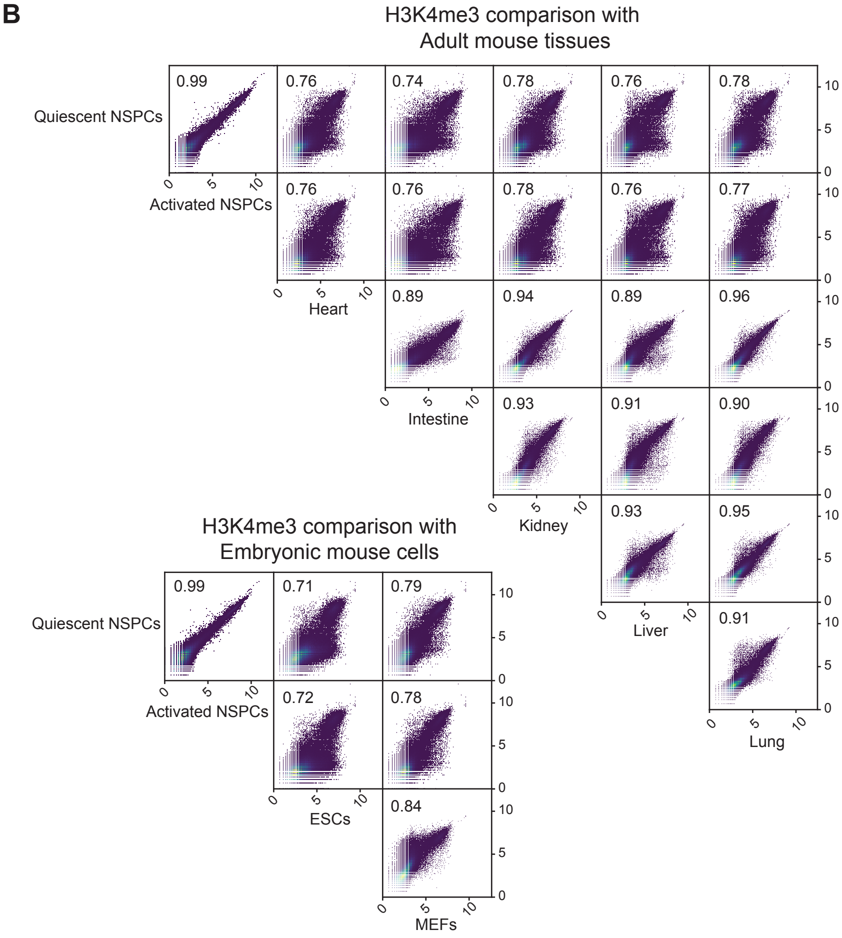
B
H3K4me3 comparison with Adult mouse tissues
Quiescent NSPCs
Activated NSPCs
Heart
Intestine
Kidney
Liver
Lung
0.99 0.76 0.74 0.78 0.76 0.78
0.76 0.76 0.78 0.76 0.77
0.89 0.94 0.89 0.96
0.93 0.91 0.90
0.93 0.95
0.91
0
5
10
H3K4me3 comparison with Embryonic mouse cells
Quiescent NSPCs
Activated NSPCs
ESCs
MEFs
0.99 0.71 0.79
0.72 0.78
0.84

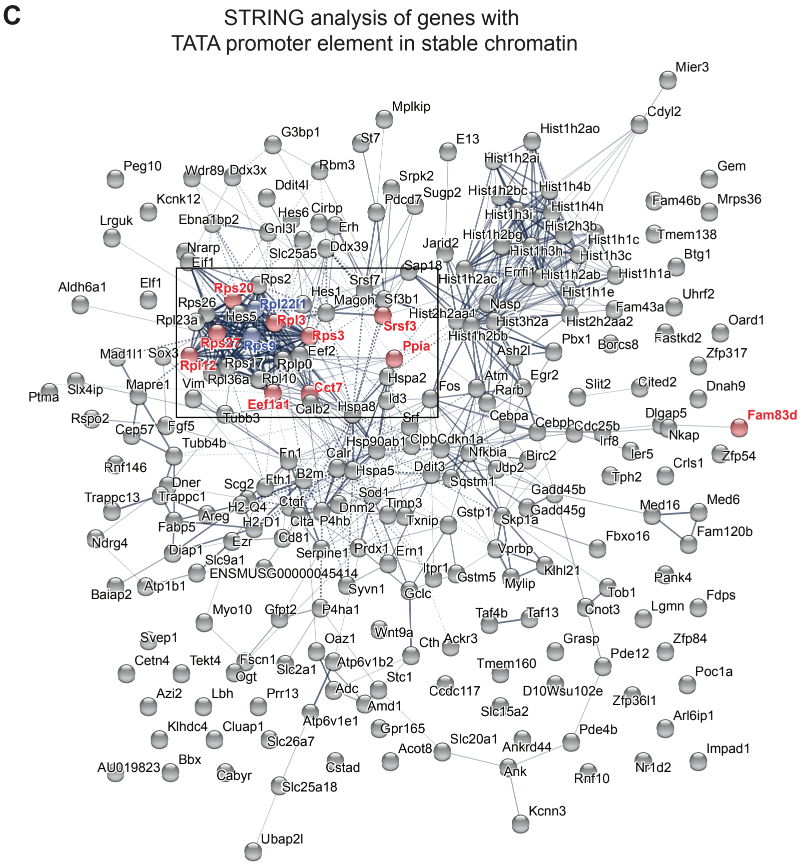
C
STRING analysis of genes with TATA promoter element in stable chromatin
Mier3
Cdyl2
Mplkip
Hist1h2ao
E13
St7
G3bp1
Hist1h2ai
Gem
Peg10
Wdr89
Ddx3x
Rbm3
Srpk2
Hist1h2bc
Hist1h4b
Mrps36
Kcnk12
Ddit4l
Sugp2
Pdcd7
Fam46b
Lrguk
Ebna1bp2
Hes6
Cirbp
Hist1h3i
Hist1h4h
Tmem138
Gnl3l
Erh
Hist1h2bg
Hist2h3b
Nrarp
Slc25a5
Ddx39
Jarid2
Hist1h1c
Btg1
Eif1
Sap18
Hist1h3h
Hist1h3c
Aldh6a1
Elf1
Rps2
Srsf7
Hist1h2ac
Errfi1
Hist1h2ab
Hist1h1a
Rps20
Hes1
Magoh
Sf3b1
Hist1h1e
Uhrf2
Rps26
Rpl22l1
Hist2h2aa1
Fam43a
Rpl23a
Hes5
Rpl3
Srsf3
Nasp
Hist3h2a
Hist2h2aa2
Oard1
Hist1h2bb
Rps3
Fastkd2
Rps27
Rps9
Ppia
Pbx1
Borcs8
Mad1l1
Sox3
Eef2
Ash2l
Zfp317
Rpl12
Rps17
Rplp0
Ptma
Slx4ip
Mapre1
Rpl10
Hspa2
Atm
Egr2
Slit2
Cited2
Dnah9
Vim
Rpl36a
Cct7
Rarb
Fos
Id3
Eef1a1
Calb2
Hspa8
Dlgap5
Fam83d
Rspo2
Cep57
Fgf5
Tubb3
Srf
Cebpa
Cebpb
Cdc25b
Nkap
Tubb4b
Hsp90ab1
ClpbCdkn1a
Irf8
Zfp54
Rnf146
Fn1
Calr
Nfkbia
Birc2
Ier5
Crls1
Dner
Scg2
Fth1
B2m
Hspa5
Ddit3
Jdp2
Tph2
Trappc13
Trappc1
Ctgf
Sqstm1
Gadd45b
H2-Q4
Sod1
Timp3
Med16
Med6
Areg
Dnm2
Gadd45g
Fabp5
H2-D1
Clta
P4hb
Txnip
Gstp1
Skp1a
Fam120b
Ndrg4
Ezr
Cd81
Fbxo16
Diap1
Serpine1
Prdx1
Ern1
Vprbp
Slc9a1
ENSMUSG00000045414
Itpr1
Klhl21
Baiap2
Atp1b1
Gstm5
Mylip
Pank4
Syvn1
Gclc
Tob1
Myo10
Gfpt2
P4ha1
Taf4b
Taf13
Cnot3
Lgmn
Fdps
Svep1
Oaz1
Wnt9a
Cth
Ackr3
Grasp
Zfp84
Cetn4
Tekt4
Fscn1
Atp6v1b2
Pde12
Slc2a1
Ogt
Tmem160
Poc1a
Stc1
Azi2
Lbh
Prr13
Adc
Ccdc117
D10Wsu102e
Zfp36l1
Amd1
Atp6v1e1
Slc15a2
Arl6ip1
Klhdc4
Cluap1
Gpr165
Pde4b
Slc26a7
Slc20a1
Ankrd44
Impad1
Acot8
AU019823
Bbx
Cabyr
Cstad
Ank
Nr1d2
Rnf10
Slc25a18
Kcnn3
Ubap2l

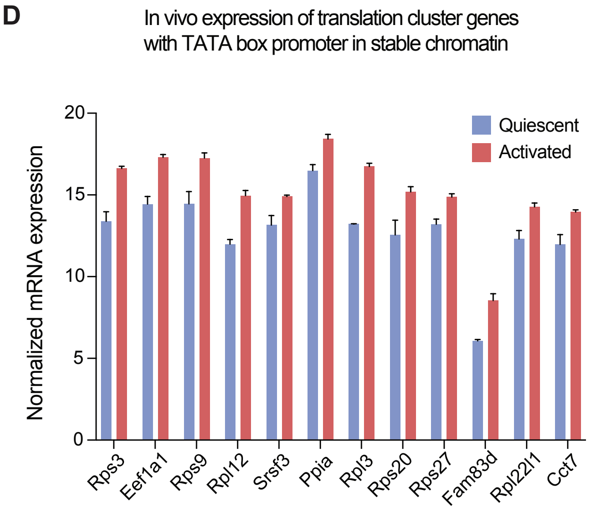
D
In vivo expression of translation cluster genes with TATA box promoter in stable chromatin
Normalized mRNA expression
0
5
10
15
20
Quiescent
Activated
Rps3
Eef1a1
Rps9
Rpl12
Srsf3
Ppia
Rpl3
Rps20
Rps27
Fam83d
Rpl22l1
Cct7

Fig. S5

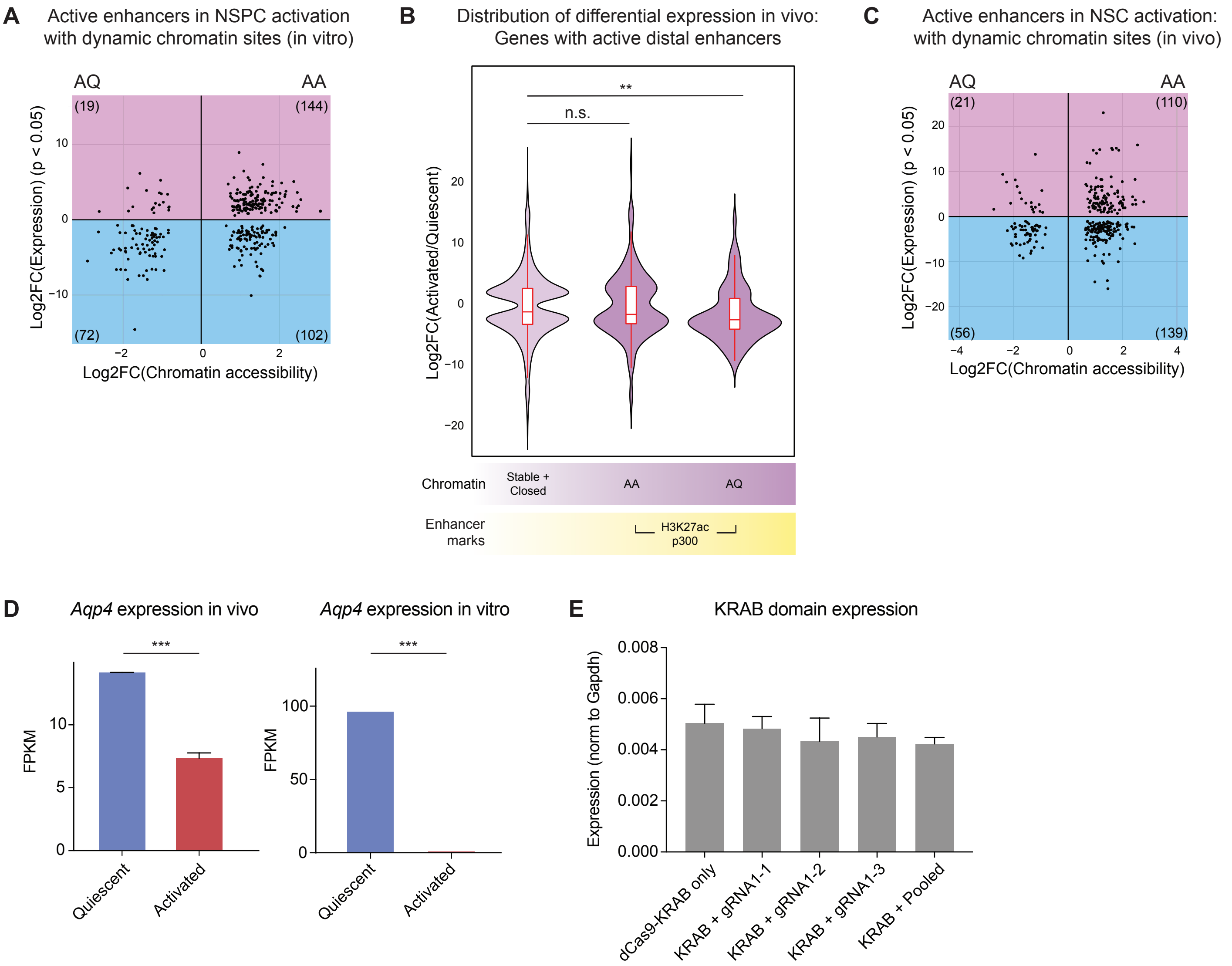
A
Active enhancers in NSPC activation
with dynamic chromatin sites (in vitro)
AQ
AA
(19)
(144)
(72)
(102)
Log2FC(Expression) (p < 0.05)
Log2FC(Chromatin accessibility)
B
Distribution of differential expression in vivo:
Genes with active distal enhancers
**
n.s.
Log2FC(Activated/Quiescent)
Chromatin
Stable + Closed
AA
AQ
Enhancer marks
H3K27ac
p300
C
Active enhancers in NSC activation:
with dynamic chromatin sites (in vivo)
AQ
AA
(21)
(110)
(56)
(139)
Log2FC(Expression) (p < 0.05)
Log2FC(Chromatin accessibility)
D
Aqp4 expression in vivo
Aqp4 expression in vitro
***
FPKM
Quiescent
Activated
E
KRAB domain expression
Expression (norm to Gapdh)
dCas9-KRAB only
KRAB + gRNA1-1
KRAB + gRNA1-2
KRAB + gRNA1-3
KRAB + Pooled

Fig. S6

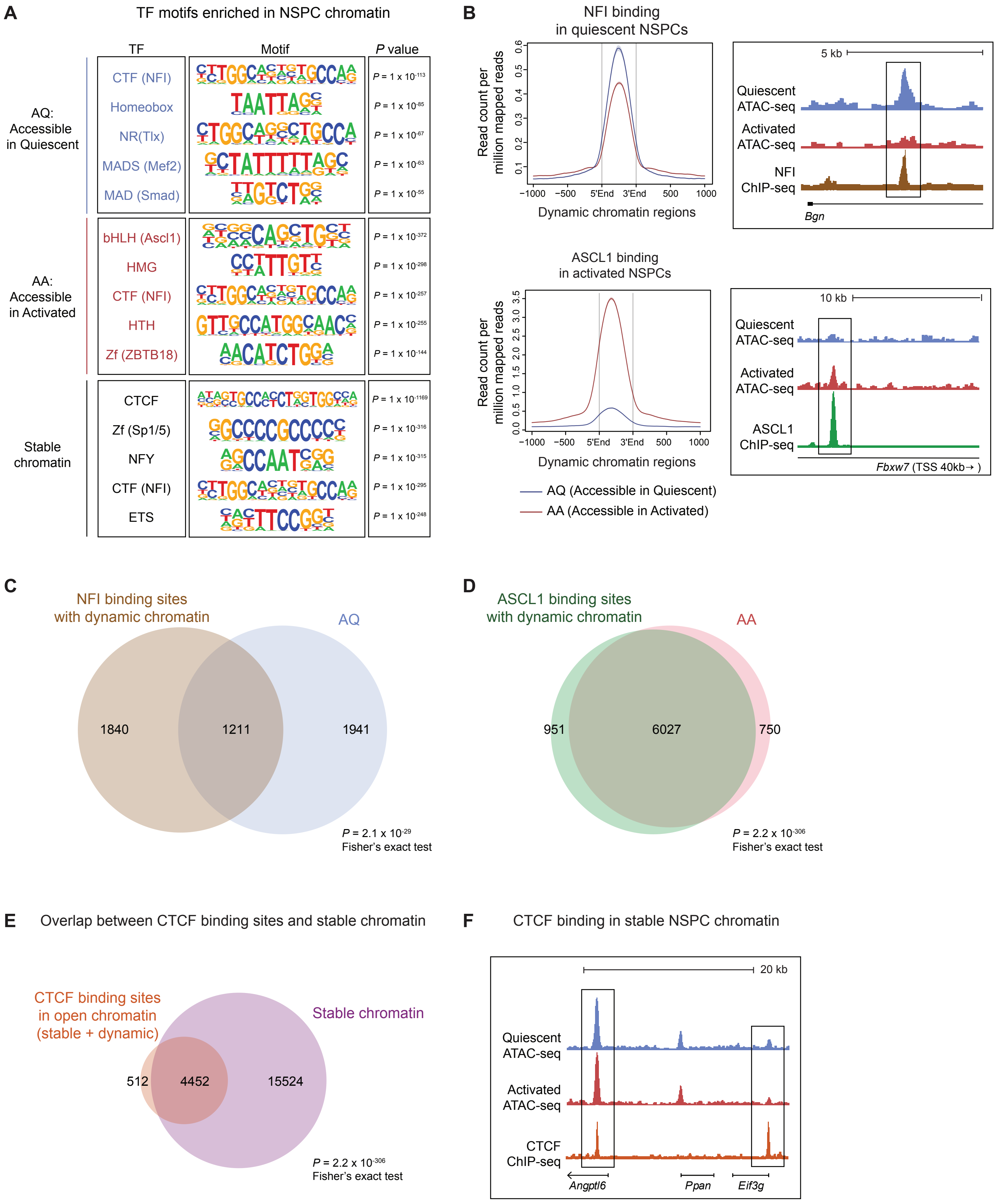

A
TF motifs enriched in NSPC chromatin
TF
Motif
P value
AQ: Accessible in Quiescent
CTF (NFI)
Homeobox
NR(Tlx)
MADS (Mef2)
MAD (Smad)
P = 1 x 10-113
P = 1 x 10-85
P = 1 x 10-67
P = 1 x 10-63
P = 1 x 10-55
AA: Accessible in Activated
bHLH (Ascl1)
HMG
CTF (NFI)
HTH
Zf (ZBTB18)
P = 1 x 10-372
P = 1 x 10-298
P = 1 x 10-257
P = 1 x 10-255
P = 1 x 10-144
Stable chromatin
CTCF
Zf (Sp1/5)
NFY
CTF (NFI)
ETS
P = 1 x 10-1169
P = 1 x 10-316
P = 1 x 10-315
P = 1 x 10-295
P = 1 x 10-248
B
NFI binding in quiescent NSPCs
Read count per million mapped reads
Dynamic chromatin regions
5 kb
Quiescent ATAC-seq
Activated ATAC-seq
NFI ChIP-seq
Bgn
ASCL1 binding in activated NSPCs
10 kb
ASCL1 ChIP-seq
Fbxw7 (TSS 40kb→)
AQ (Accessible in Quiescent)
AA (Accessible in Activated)
C
NFI binding sites with dynamic chromatin
AQ
1840
1211
1941
P = 2.1 x 10-29
Fisher's exact test
D
ASCL1 binding sites with dynamic chromatin
AA
951
6027
750
P = 2.2 x 10-306
Fisher's exact test
E
Overlap between CTCF binding sites and stable chromatin
CTCF binding sites in open chromatin (stable + dynamic)
Stable chromatin
512
4452
15524
P = 2.2 x 10-306
Fisher's exact test
F
CTCF binding in stable NSPC chromatin
20 kb
Quiescent ATAC-seq
Activated ATAC-seq
CTCF ChIP-seq
Angptl6
Ppan
Eif3g

Fig. S7

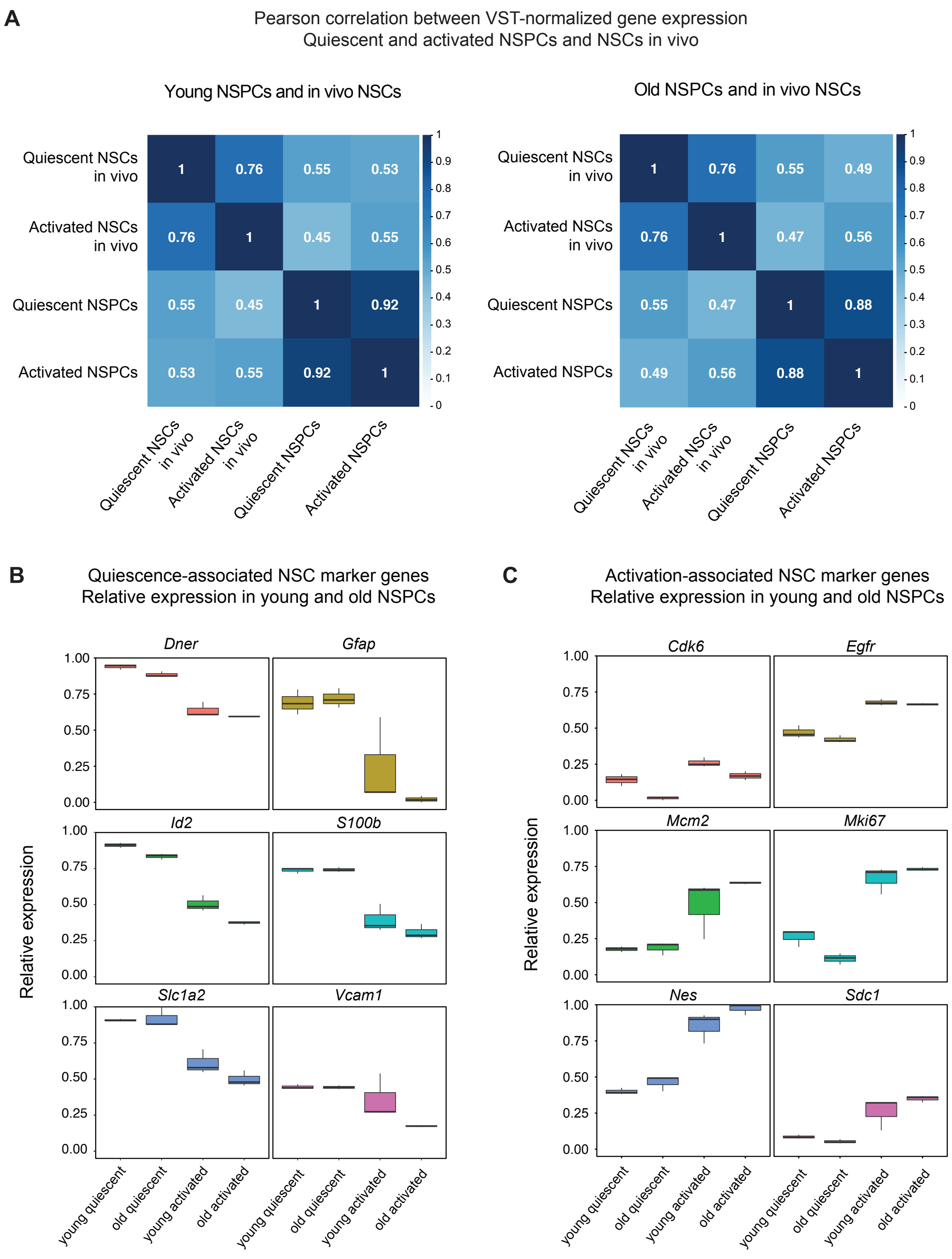
A
Pearson correlation between VST-normalized gene expression
Quiescent and activated NSPCs and NSCs in vivo
Young NSPCs and in vivo NSCs
Old NSPCs and in vivo NSCs
Quiescent NSCs in vivo
Activated NSCs in vivo
Quiescent NSPCs
Activated NSPCs
B
Quiescence-associated NSC marker genes
Relative expression in young and old NSPCs
Dner
Gfap
Id2
S100b
Slc1a2
Vcam1
C
Activation-associated NSC marker genes
Relative expression in young and old NSPCs
Cdk6
Egfr
Mcm2
Mki67
Nes
Sdc1
Relative expression
young quiescent
old quiescent
young activated
old activated

Fig. S8

Reactome pathway analysis:
Age-associated loss in chromatin accessibility in quiescent NSPCs

Parent Pathway	Pathway Name and Identifier	*P*-value (FDR = n.s.)	Genes
Disease of metabolism	Defective EXT1 causes exostoses 1, TRPS2 and CHDS (R-HSA-3656253)	0.01	*Sdc2;Gpc5;Agrn;Gpc6*
	Defective EXT2 causes exostoses 2 (R-HSA-3656237)	0.01	
	Defective B3GALT6 causes EDSP2 and SEMDJL1 (R-HSA-4420332)	0.03	
	Defective B4GALT7 causes EDS, progeroid type (R-HSA-3560783)	0.03	
	Defective B3GAT3 causes JDSSDHD (R-HSA-3560801)	0.03	
Metabolism	Alanine metabolism (R-HSA-8964540)	0.04	*Gpt2*
RNA Pol II transcription	FOXO-mediated transcription of cell cycle genes (R-HSA-9617828)	0.01	*Btg1;Pcbp4;Gadd45a*
	TFAP2 (AP-2) family regulates transcription of other transcription factors (R-HSA-8866906)	0.03	*Pitx2*
	RUNX2 regulates genes involved in cell migration (R-HSA-8941332)	0.04	*Cbfb;Itgbl1*
Innate immune system	Alternative complement activation (R-HSA-173736)	0.04	*C3;Cfp*
	Toll Like Receptor 3 (TLR3) Cascade (R-HSA-168164)	0.04	*Cap1;Alcam;Ppp2r1a;Ripk2; Ube2d3;Tab3;Birc2;Elk1;Map2k7*
Signal transduction	NOTCH3 Intracellular Domain Regulates Transcription (R-HSA-9013508)	0.04	*Maml2;Fabp7;Hey1*
