# Supplementary material for: Changing and stable chromatin accessibility supports transcriptional overhaul during neural stem cell activation and is altered with age
Source: Aging Cell. 2021 Oct 23;20(11):e13499. doi: 10.1111/acel.13499 (PMC8590101; doi:10.1111/acel.13499)
Supplement: Supplementary file 12 — Supplementary Material [file ACEL-20-e13499-s008.docx]

**Changing and stable chromatin accessibility supports transcriptional overhaul during neural stem cell activation and is altered with age**

Sun Y. Maybury-Lewis^1^, Abigail K. Brown^1^, Mitchell Yeary^1^, Anna Sloutskin^2^, Shleshma Dhakal^1^, Tamar Juven-Gershon^2^, and Ashley E. Webb^1,3,4,^*

^1^Department of Molecular Biology, Cell Biology, and Biochemistry, Brown University, Providence, RI 02912, USA.

^2^The Mina and Everard Goodman Faculty of Life Sciences, Bar-Ilan University, Ramat Gan, Israel.

^3^Carney Institute for Brain Science, Brown University, Providence, RI 02912, USA.

^4^Center on the Biology of Aging, Brown University, Providence, RI 02912, USA.

*Correspondence: Ashley_Webb@brown.edu

Abbreviated title: Chromatin states in neural stem cell aging

**Supporting Information**

**Fig. S1. Activated and BMP4-induced quiescent NSPCs express NSC markers and are distinct from other cell types in their open chromatin profiles.** (A) Percentage of EdU-positive cells in activated, quiescent, and reactivated states. (n = 3, ****P* < 0.001, Student’s t-test, mean ± SD). (B) SOX2 and NESTIN expression in activated, quiescent, and reactivated NSPCs from young (top) and old (bottom) brains. Green, SOX2; red, NESTIN. Scale bar represents 100 microns. (C) Pearson correlation of ATAC-seq signals among quiescent and activated NSPCs and other mouse tissues (lung, liver, intestine, kidney, stomach). (D) UCSC genome browser snapshots of *Olig2* (left) and *Fgf1* (right) loci with quiescent and activated ATAC-seq, H3K27ac and p300 ChIP-seq tracks marking active enhancers.

**Fig. S2. Dynamic chromatin regions are associated with differential expression of genes between quiescent and activated states *in vitro*.** (A) Percentages of differentially expressed genes NS5-derived neural progenitors *in vitro* that contain dynamic (AQ or AA) chromatin sites, or both. (B) Comparison of differential expression of genes with chromatin showing no change (constitutively accessible and closed chromatin) and AA and AQ dynamic chromatin (****P* < 0.001, Wilcoxon rank sum test). (C) Scatterplot depicting fold change in chromatin accessibility versus fold change in associated gene expression *in vitro* (FDR < 0.05). Each dot is a dynamic site that is associated with a differentially expressed gene, and the total number of sites in each quadrant is shown in parentheses. Downregulated genes are enriched in AQ sites (*P* = 3.2 x 10^-12^, Fisher’s exact test). (D) Venn diagrams depicting overlap of differentially expressed genes across RNA-seq datasets (*in vivo* NSCs, *in vitro* NS5 cells, cultured primary NSPCs).

**Fig. S3. The chromatin accessibility profile of reactivated NSPCs resembles that of activated NSPCs.** (A) Pearson correlation coefficient r (0.87 in activated, 0.91 in quiescent, 0.95 in reactivated) in ATAC-seq signals between biological replicates in activated, quiescent, and reactivated conditions. (B) Principal component analysis of biological replicates in activated, quiescent, and reactivated ATAC-seq. (C) Genomic distribution of all open chromatin regions in reactivated NSPCs. (D) Overlaps among open chromatin regions in reactivated NSPCs and stable or dynamic (AA or AQ) chromatin regions in quiescent and activated NSPCs. (E) Comparison of differential expression *in vivo* (fold change) between genes with chromatin with no change, AA chromatin, AQ chromatin, AA chromatin accessible in reactivated NSPCs (AR:AA), and AQ chromatin accessible in reactivated NSPCs (AR:AQ) (****P* < 0.001, ***P* < 0.01, Wilcoxon rank sum test).

**Fig. S4. Constitutively accessible chromatin regions in NSPCs are enriched with cell type-specific active promoter marks and specific core promoter elements.** (A) Overlaps between stably open chromatin sites and H3K4me3-enriched sites in quiescent NSPCs (top) (*P* = 2.0 x 10^-81^, Fisher’s exact test) and activated NSPCs (bottom) (*P* = 3.3 x 10^-125^, Fisher’s exact test). (B) Pearson correlation coefficient r among H3K4me3 ChIP-seq signals from quiescent and activated NSPCs and published adult mouse tissues (top) and embryonic mouse cells (bottom). Correlation coefficients are shown in upper left corner of each plot. (C) STRING analysis of all genes with the TATA box motif in constitutively accessible promoters. Genes upregulated in aNSCs *in vivo* compared to quiescent cells with the TATA box motif within biologically relevant positions (-40/-41 to -13/-14 bp from TSS) are shown in red. Genes containing both the TATA box and TCT motifs (-11 to +16 bp from TSS) in their promoters are shown in blue. The top quartile of upregulated genes are clustered together, consisting of ribosomal and translation-related genes (boxed). (D) Normalized mRNA expression values from quiescent and activated *in vivo* NSCs RNA-seq. A cluster of ribosomal and translation-associated genes are consistently upregulated in NSC activation, and harbor the TATA box motif in their core promoters (-40/-41 to -13/-14 bp from TSS).

**Fig. S5. Active enhancers in dynamic chromatin and their association to differential gene expression.** (A) Scatterplot depicting fold change in chromatin accessibility at active enhancers versus fold change in associated gene expression in NS5-derived progenitors *in vitro* (FDR < 0.05). Each dot is a dynamic site with active enhancer marks associated with a differentially expressed gene, and the total number of sites in each quadrant is shown in parentheses. (B) Comparison of differential expression in vivo between genes with no changes in chromatin and genes with active enhancers in dynamic chromatin (***P* < 0.01, Wilcoxon rank sum test). (C) Scatterplot showing fold change in chromatin accessibility versus fold change in associated gene expression *in vivo* (FDR < 0.05). Display is as described in (A). (D) Differential expression of *Aqp4* in quiescent and activated NSCs *in vivo* (left) and cultured NS5 cells (right). *Aqp4* is significantly upregulated in quiescent cells (***FDR < 0.001). (E) RT-qPCR analysis of the KRAB repressor domain in NSPCs infected with lentiviruses carrying dCas9-KRAB and gRNAs (n = 3 experiments, mean ± SD).

Fig. S6. Dynamic chromatin sites are bound by NFI and ASCL1, major regulators of NSPCs, while stable chromatin sites are enriched for CTCF. (A) Summary of Homer motif analysis in AQ, AA, and constitutively accessible chromatin regions. The top 5 most highly enriched motifs are shown for each chromatin state. (B) Pan-NFI (top) and ASCL1 (bottom) ChIP-seq signals from quiescent and activated NSPCs plotted in 5’ to 3’ direction in dynamic chromatin regions (left). UCSC genome browser snapshots of a representative AQ chromatin site with NFI binding (top) and an AA chromatin site with ASCL1 binding (bottom) are shown on the right. (C) Overlap of NFI binding with AQ chromatin sites (*P* = 2.1 x 10^-29^, Fisher’s exact test). (D) Overlap of ASCL1 binding with AA chromatin sites (*P* = 2.2 x 10^-306^, Fisher’s exact test). (E) Overlaps between constitutively accessible chromatin and CTCF binding open chromatin sites (*P* = 2.2 x 10^-306^, Fisher’s exact test). (F) UCSC genome browser snapshot examples of constitutively accessible chromatin binding CTCF.

**Fig. S7. Changes in chromatin accessibility are detected in quiescent NSPCs with age.** (A) Pearson correlation r values between normalized gene expression in young cultured NSPCs and *in vivo* NSCs (left) and old cultured NSPCs and *in vivo* NSCs (right). (B) Normalized expression levels of quiescence-associated NSC marker genes from young and old NSPCs RNA-seq. (C) Normalized expression levels of activation-associated NSC marker genes from young and old NSPCs RNA-seq.

**Fig. S8. Summary of Reactome Pathway analysis in genes with differential accessibility in young and old quiescent NSPCs** Out of 672 genes associated with differentially accessible sites, 347 genes were found in the Reactome Knowledgebase (*P* < 0.05).

**Table S1.** Read numbers for ATAC-seq libraries and peak calling results.

**Table S2.** Summary of DiffBind differential accessibility analysis.

**Table S3.** Summary of GREAT gene assignments to dynamic and stable chromatin sites.

**Table S4.** Read numbers for H3K4me3 ChIP-seq libraries and peak calling results.

**Table S5.** Complete tables of Ingenuity Pathway Analysis (IPA) results.

**Table S6.** Complete tables of core promoter element analysis (ElemeNT) results.

**Table S7.** Oligonucleotide sequences for lentivirus experiments.

**Table S8.** Complete tables of iRegulon network inference results.

**Table S9**. Summary of 20-month-old ATAC-seq libraries and analyses.

**Table S10**. Quiescent and activated NSPCs RNA-seq from young and old mouse brains.
